# A non-lethal malarial infection results in reduced drug metabolizing enzyme expression and drug clearance in mice

**DOI:** 10.1186/s12936-019-2860-5

**Published:** 2019-07-12

**Authors:** Sylvie M. Mimche, Choon-myung Lee, Ken H. Liu, Patrice N. Mimche, R. Donald Harvey, Thomas J. Murphy, Beatrice A. Nyagode, Dean P. Jones, Tracey J. Lamb, Edward T. Morgan

**Affiliations:** 1 Department of Pharmacology and Chemical Biology, Emory University School of Medicine, Atlanta, GA 30322 USA; 2 Division of Pulmonary, Allergy and Critical Care Medicine, Department of Medicine, Emory University School of Medicine, Atlanta, GA 30322 USA; 3 Division of Infectious Diseases, Department of Pediatrics, Emory University School of Medicine, Atlanta, GA 30322 USA; 4 Department of Hematology and Medical Oncology, Winship Cancer Institute, Emory University, Atlanta, GA 30322 USA

**Keywords:** *Plasmodium chabaudi chabaudi*, Drug metabolism, Cytochrome P450, Liver, Gene expression

## Abstract

**Background:**

Given the central importance of anti-malarial drugs in the treatment of malaria, there is a need to understand the effect of *Plasmodium* infection on the broad spectrum of drug metabolizing enzymes. Previous studies have shown reduced clearance of quinine, a treatment for *Plasmodium* infection, in individuals with malaria.

**Methods:**

The hepatic expression of a large panel of drug metabolizing enzymes was studied in the livers of mice infected with the AS strain of *Plasmodium chabaudi chabaudi*, a nonlethal parasite in most strains of mice with several features that model human *Plasmodium* infections. C57BL/6J mice were infected with *P. chabaudi* by intraperitoneal injection of infected erythrocytes and sacrificed at different times after infection. Relative hepatic mRNA levels of various drug metabolizing enzymes, cytokines and acute phase proteins were measured by reverse transcriptase-real time PCR. Relative levels of cytochrome P450 proteins were measured by Western blotting with IR-dye labelled antibodies. Pharmacokinetics of 5 prototypic cytochrome P450 substrate drugs were measured by cassette dosing and high-resolution liquid chromatography-mass spectrometry. The results were analysed by MANOVA and post hoc univariate analysis of variance.

**Results:**

The great majority of enzyme mRNAs were down-regulated, with the greatest effects occurring at the peak of parasitaemia 8 days post infection. Protein levels of cytochrome P450 enzymes in the Cyp 2b, 2c, 2d, 2e, 3a and 4a subfamilies were also down-regulated. Several distinct groups differing in their temporal patterns of regulation were identified. The cassette dosing study revealed that at the peak of parasitaemia, the clearances of caffeine, bupropion, tolbutamide and midazolam were markedly reduced by 60–70%.

**Conclusions:**

These findings in a model of uncomplicated human malaria suggest that changes in drug clearance in this condition may be of sufficient magnitude to cause significant alterations in exposure and response of anti-malarial drugs and co-medications.

**Electronic supplementary material:**

The online version of this article (10.1186/s12936-019-2860-5) contains supplementary material, which is available to authorized users.

## Background

Malaria is responsible for approximately 0.5 million deaths every year, mostly affecting pregnant women and children under 5 years of age [1]. The development of drug resistance against standard anti-malarial therapies is an ongoing problem and this has necessitated the use of combination therapies in certain areas of the world. Quinine has been used previously to treat symptoms of malaria, and many studies have found that the clearance of quinine is reduced in humans with uncomplicated *Plasmodium falciparum* malaria [2–6]. The magnitude of the effect is greater in patients with cerebral malaria and is also correlated with the degree of parasitaemia [4, 7]. As such, understanding the effect of disease on metabolism of anti-malarial drugs is important to ensure that dosing regimens are appropriate, efficacious and do not unduly foster conditions that may select for drug-resistance.

Data on the effects of *Plasmodium* infection on drug metabolizing enzymes (DMEs) are sparse. However, the evidence collectively suggests that *Plasmodium* infection has a significant effect on the regulation of the cytochrome P450 (CYP) family of DMEs. CYP3A enzymes are mainly responsible for quinine clearance via its 3-hydroxylation [8], indirectly suggesting that expression of CYP3A may be reduced in *Plasmodium*-infected individuals. A study of caffeine clearance in adult male patients with severe falciparum malaria found significantly reduced clearance compared to the convalescent phase [9] and another study found significantly lower caffeine clearance in children with malaria compared with healthy controls [10]. Although another study found no effect [11], the ratio of paraxanthine to caffeine, an index of CYP1A2 activity, was affected more than total caffeine clearance [10, 11]. Studies in rats [12–14] and mice [15–18] infected with the rodent *Plasmodium* parasites *Plasmodium berghei* or *Plasmodium yoelii*, have demonstrated reductions in hepatic microsomal metabolism of various cytochrome P450 (P450) substrates. One study found that in rats infected with the ANKA strain of *P. berghei,* total hepatic microsomal P450 was reduced by 56%, and CYP3A2 protein by 32%, whereas CYP2E1 protein was unaffected. In agreement with these findings, testosterone 6β-hydroxylation (CYP3A2) was reduced by 41% and chlorzoxazone hydroxylation (CYP2E1) was unchanged [19]. Cyp3a11, Cyp1a2 and Cyp2e1 mRNAs were also down-regulated by more than 80% in *P. berghei*-infected animals which were accompanied by prolonged midazolam sleeping time and moderate increases in chlorzoxazone plasma concentrations [18]. In pregnant mice infected with *P. berghei*, a 65% down-regulation of Cyp3a11 mRNA was reported, together with both down-and up-regulation of different members of the ABC family of drug transporters [20].

Understanding the scope of malarial effects on drug metabolism, as well as elucidating the mechanisms leading to such changes, is necessary for optimal chemotherapeutic treatment of malaria. Previously published studies have used *P. berghei* ANKA infections of C57BL/6 mice. *Plasmodium berghei* ANKA is an accepted model of cerebral malaria with mice dying between 7 and 10 days post-infection from cerebral symptoms that partly resemble human cerebral malaria. As such, results are more relevant to the modulation of DMEs in *Plasmodium*-infected individuals at the severe end of the malaria spectrum. Given that the human studies described above found significant changes in drug clearance in people with uncomplicated malaria, it is important to study DMEs in a rodent model of non-cerebral malaria. *Plasmodium chabaudi chabaudi* AS strain is a non-lethal infection in C57BL/6 mice. *Plasmodium chabaudi* AS has several characteristics similar to human *P. falciparum* infections [21], and mice experience temperature dysregulation, anaemia and weight loss before controlling the infection. There is only one limited report describing changes in hepatic microsomal drug metabolizing activities in *P. chabaudi*-infected mice [17]. Therefore, this study quantified DME expression and in vivo activity of DMEs during *P. chabaudi* infection.

## Methods

### Animals and *Plasmodium* infection

All animal procedures were reviewed and approved by the Institutional Animal Care and Use Committee of Emory University. Female C57BL/6J mice (aged 6–8 weeks old) from The Jackson Laboratory (Bar Harbor, ME) were housed under standard conditions and were fed a normal diet (LabDiet, St. Louis, MO; chow 5001) with water ad libitum. Mice were monitored for general health, weight loss and anaemia throughout infections to ensure they did not reach IACUC endpoints. *Plasmodium chabaudi* AS infections were initiated by intraperitoneal (ip) injection of 10^5^ infected red blood cells (iRBCs) obtained from infected donor mice and suspended in Kreb’s saline. At the end of the infection period, mice were euthanized by CO_2_ asphyxiation, and livers were dissected, rinsed in cold 1.15% KCl, then weighed, portioned, flash-frozen and stored at − 80 °C for subsequent RNA, S9 fraction and microsome preparation.

For the first part of this study on DME mRNA expression, mice were sacrificed 6, 8 or 12 days after infection. Data were pooled from two identical experiments. There were 4 controls and 8 infected animals in the 6- and 12-day post-infection groups, whereas there were 6 controls and 10 infected mice in the 8-day cohorts. For determination of relative levels of hepatic P450 proteins, a separate experiment was conducted with 3 mice per group. The mice used for the pharmacokinetic experiment (n = 5 per group) also comprised a separate experiment.

### Parasitaemia and liver burden measurements

Parasitaemia was measured by counting iRBCs in 300–500 total red blood cells in Giemsa-stained blood smears as described previously [22]. Liver burden of *P. chabaudi* AS was assessed via the levels of *P. chabaudi* merozoite surface protein-1 (MSP1) mRNA, measured by real time reverse-transcriptase polymerase chain reaction (RT-qPCR) (primer sequences are in Additional file 1: Table S1).

### Liver microsome preparation

Hepatic S9 factions and microsomes were prepared by centrifugation as described in a previous publication [23]. A bicinchoninic acid protein assay kit (Thermo Fisher Scientific, Inc., Rockport, IL) was used to measure protein concentrations, with bovine serum albumin as the standard.

### Western blotting

Relative levels of P450 or Nos2 proteins in mouse hepatic S9 fractions or microsomes were measured by SDS-polyacrylamide gel electrophoresis, Western blotting and infrared detection. For Cyp4a proteins only, microsomes were used to improve the signal to noise ratio. Equal amounts of S9 or microsomal protein were resolved on SDS-PAGE and blotted on nitrocellulose paper (Bio-Rad, Hercules, CA). The antibodies used, their sources and dilutions are listed in Additional file 2: Table S2. The P450 antibodies have been previously described [23]. Glyceraldehyde-3-phosphate dehydrogenase (GAPDH, 1:10,000; Millipore, Temecula, CA) was used as a loading control. GAPDH antibodies were included in the primary incubations with the P450 or Nos2 antibodies. After washing, the blots were incubated with IRDye^®^ 680RD Goat anti-Rabbit IgG and IRDye^®^ 800CW Goat anti-Mouse IgG (1:10,000 dilution) (LI-COR Biosciences, Lincoln, NE) for 1 h and analysed with an Odyssey^®^ Fc Imaging System (LI-COR Biosciences) to detect fluorescence signals. Image Studio™ software (LI-COR Biosciences) was used to measure fluorescence intensity, and P450 or Nos2 signals were normalized to Gapdh signal intensities.

### RT-qPCR and primer sequences

RNA-Bee isolation reagent (Tel-Test Inc., Friendswood TX) was used to prepare total liver RNA. RNA concentration was measured spectrophotometrically at 260 nm, and its purity and integrity were confirmed by formaldehyde-agarose gel electrophoresis followed by visualization with ethidium bromide. Total RNA was reverse-transcribed using a High Capacity cDNA reverse transcription kit (Invitrogen, Carlsbad, CA). Relative levels of specific mRNAs in the samples relative to 18S RNA were measured by RT-qPCR as described previously [24, 25]. Melting curves were checked routinely to ensure primer specificity. Primers used for RT-qPCR measurements are listed in Additional file 1: Table S1, the majority of which have been used previously in this laboratory [23, 24].

### Pharmacokinetics

A cassette dosing pharmacokinetic study was carried out via the method described by Scheer et al. [26]. Mice (control and infected, 8 days post infection, n = 5) were administered a five-drug cocktail containing caffeine, bupropion, tolbutamide, bufuralol, and midazolam in saline by oral gavage, to give final doses of 5, 30, 5, 10, and 3 mg/kg, respectively. Blood (10 µL) was collected from the transected tip of the tails at 10, 20, 40, 60, 120, 240, 360, and 480 min after administration, and transferred into microcentrifuge tubes containing heparin (10 µL). 95 µL of acetonitrile containing a mixture of isotopically labelled internal standards including [3^13^C]-caffeine was added to the samples, vortexed vigorously for 20 s, and centrifuged at 12,000*g* for 5 min. The supernatants were transferred into HPLC sample vials for UHPLC/HRMS using a Dionex Ultimate 3000 UHPLC system coupled to a Thermo Scientific High-Field Q-Exactive for High-Resolution Mass Spectrometry (HRMS) with positive electrospray ionization (ESI+).

Hydrophilic interaction chromatography (HILIC) was performed using a Waters XBridge 2.5 μM BEH Amide 2.1 × 50 mm column (Waters, Ireland) with mobile phases of A: LCMS grade water (Sigma-Aldrich), B: LCMS grade Acetonitrile (Sigma-Aldrich), and C: 2% LCMS grade Formic Acid (Sigma-Aldrich) in LCMS grade water. Using a flow rate of 0.350 mL/min, starting mobile phase conditions were 22.5% A, 75% B, 2.5% C. These conditions were held from 0 to 1.5 min before a linear gradient from 1.5 to 4.0 min to 77.5% A, 20% B, 2.5% C, and held for another minute. Following completion of the 5-min gradient cycle, the column was returned to initial mobile phase conditions for re-equilibration for 5.5 min prior to the subsequent injection.

HRMS analysis was performed using a scan range of 85–1275 *m*/*z* at 120k resolution (4 full scans/s) with an AGC target of 1e6 and maximum injection time of 100 ms. Calibration curves were prepared from authentic drug standards for quantification. HRMS analysis was performed in a targeted fashion, with accurate masses corresponding to an [M+H] ion for drugs manually extracted (± 2 ppm) and integrated for quantification using Xcalibur Qual Browser Software (Thermo Scientific). The following accurate masses were used: caffeine (M+H: 195.0877 *m*/*z*), bufuralol (M+H: 262.1802 *m*/*z*), bupropion (M+H: 240.1150 *m*/*z*), midazolam (M+H: 326.0855 *m*/*z*), tolbutamide (M+H: 271.1111 *m*/*z*). No background was detected in a non-experimental/non-spiked blood sample for any of these masses. MS2 spectra for cocktail drugs was collected using parallel reaction monitoring (PRM) (HCD: 35 eV) for standards and pooled reference quality control (QC) samples.

Three technical replicate injections for each sample were injected to assess method precision and injection reproducibility. Peak areas from triplicate analysis were averaged, with median technical replicate CV% for each drug being less than 8%. A pooled reference QC sample containing a mixture of experimental samples was prepared, split into four vials, and analysed twice at the beginning of the run (before and after standard materials), once in the middle of the run, and then at the end of the run. Over the entire run, the QC samples showed consistent peak areas for the same analyte in the QC sample: tolbutamide CV% < 7%, caffeine CV% < 10%, bupropion CV% < 12%, bufuralol CV% < 6%, and Midazolam CV% < 17%. This data validates the precision of the method, and the use of reference standardization [27], for calculating drug concentrations in experimental samples.

Sample concentrations were calculated by reference standardization. Briefly, authentic drug standards were used to determine the concentration of each drug in the pooled reference (mixture of experimental samples). Each standard curve (Additional file 3: Fig. S1) bracketed the highest and lowest peak areas observed in experimental samples and established a linear response over all peak areas observed in experimental samples. Drug concentrations in the pooled reference sample were then used to calculate concentrations for all experimental samples. Limits of quantification (technical replicate CV > 30% or the lowest concentration standard if replicate CV < 30%) for each drug are as follows: tolbutamide: 144 nM, linear range 144 nM–37 μM, pooled reference 15.4 μM; caffeine: 100 nM, linear range 100 nM–25.7 μM, pooled reference 5.8 μM; bupropion: 163 nM, linear range 163 nM–41.7 μM, pooled reference 910 nM; midazolam: 15 nM, linear range 15 nM–3.8 μM, pooled reference 261 nM; Bufuralol: 15 nM, linear range 15 nM–3.8 μM, pooled reference 370 nM. These limits were determined by the use of matrix matched mouse blood collected from control mice.

Plasma concentration-time data for each CYP probe drug in the cocktail in control and infected mice were analysed for pharmacokinetic parameters with Phoenix^®^ WinNonlin^®^ software, version 6.0 (Certara USA, Princeton, NJ) using noncompartmental methods.

### Statistical analysis

For analysis of the gene expression data, the value obtained from each target gene was normalized to 18S RNA and relative levels were calculated using the delta delta Ct method [28]. Because target gene to 18S RNA ratios varied from analysis to analysis, fold-to-control baseline corrected values were calculated by dividing each ratio by the mean of the control treatment ratios. To test whether any mRNAs were affected by infection, multivariate analysis of variance on the gene expression matrix was performed as an omnibus test using the MANOVA function in the base package in R. To identify specific mRNAs affected by infection, post hoc univariate analysis of variance for the main effect of treatment (infection) was performed following a positive MANOVA result. The univariate ANOVA results were interpreted after applying a Bonferroni correction factor of 34 for the multiple post hoc comparisons.

MANOVA analyses and Bonferroni corrections were also performed on the Western blot measurements of protein expression, and to determine the effects of infection on pharmacokinetic parameters.

## Results

### Time course of parasitaemia and liver burden of *Plasmodium chabaudi AS*

**Fig. 1:**
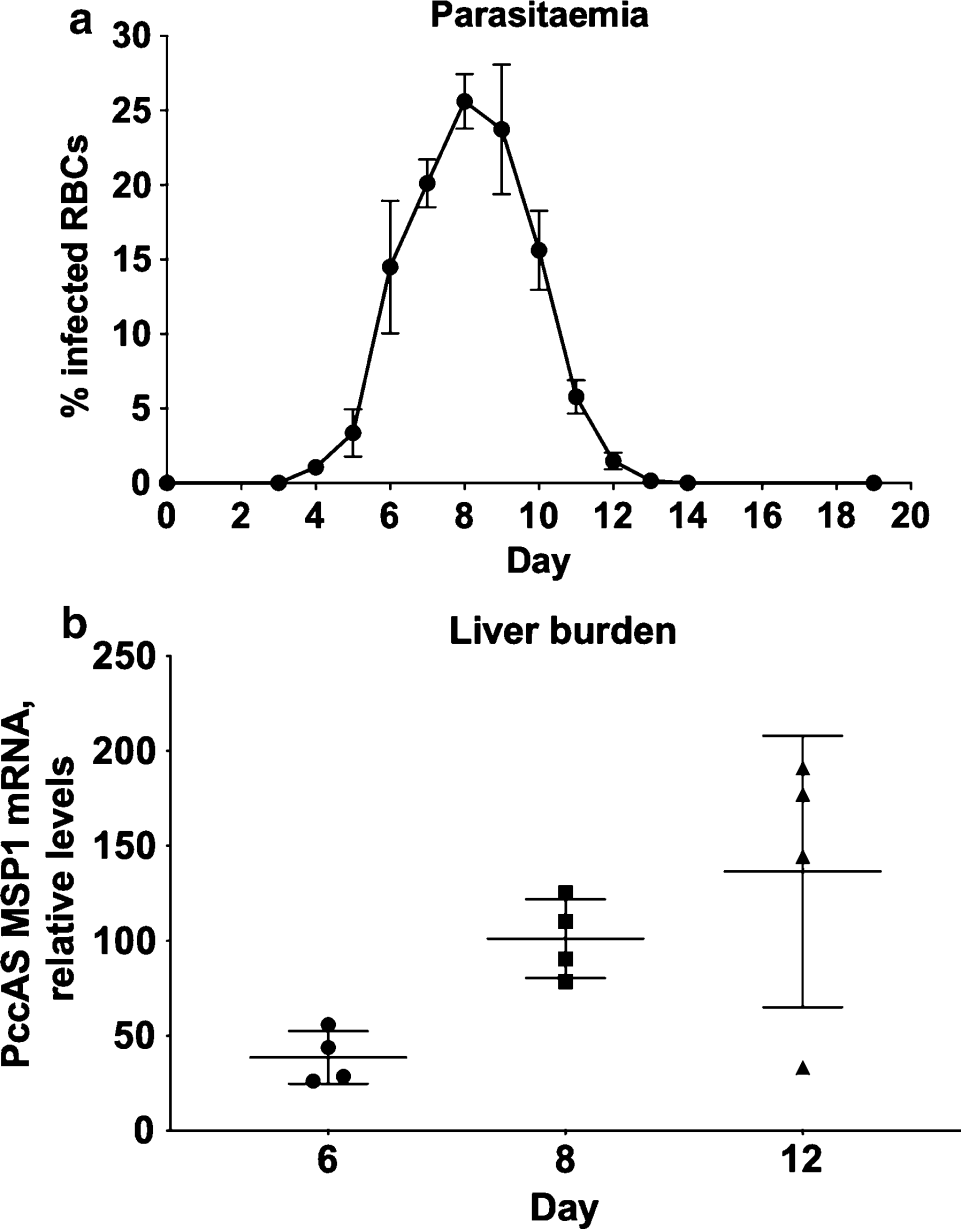
Burdens of *P. chabaudi* in the blood and livers of infected mice. **a** Percent parasitaemia in *P. chabaudi* -infected mice over time (n = 8). **b** Relative levels of *P. chabaudi* AS in the liver determined by RT-qPCR, n = 4. Values are mean ± SD

As reported in previous work [22], iRBCs were first detected on day 5, reached a peak of 25% on day 8, and declined to < 1% at 13 days of infection (Fig. 1a, Additional file 4). The liver burden of *P. chabaudi*, measured by qPCR analysis of MSP1 mRNA, rose steadily from 6 to 12 days post infection (Fig. 1b). The time points of 6, 8 and 12 days were selected for analysis of DMEs to reflect the growth, peak and contraction of infection time points. Note that the liver burden of parasites largely derives from iRBCs sequestered in the sinusoids, not intrahepatocellular parasites which cannot be rederived from an infection initiated with iRBCs.

### Hepatic expression of drug metabolizing enzyme mRNAs

**Fig. 2:**
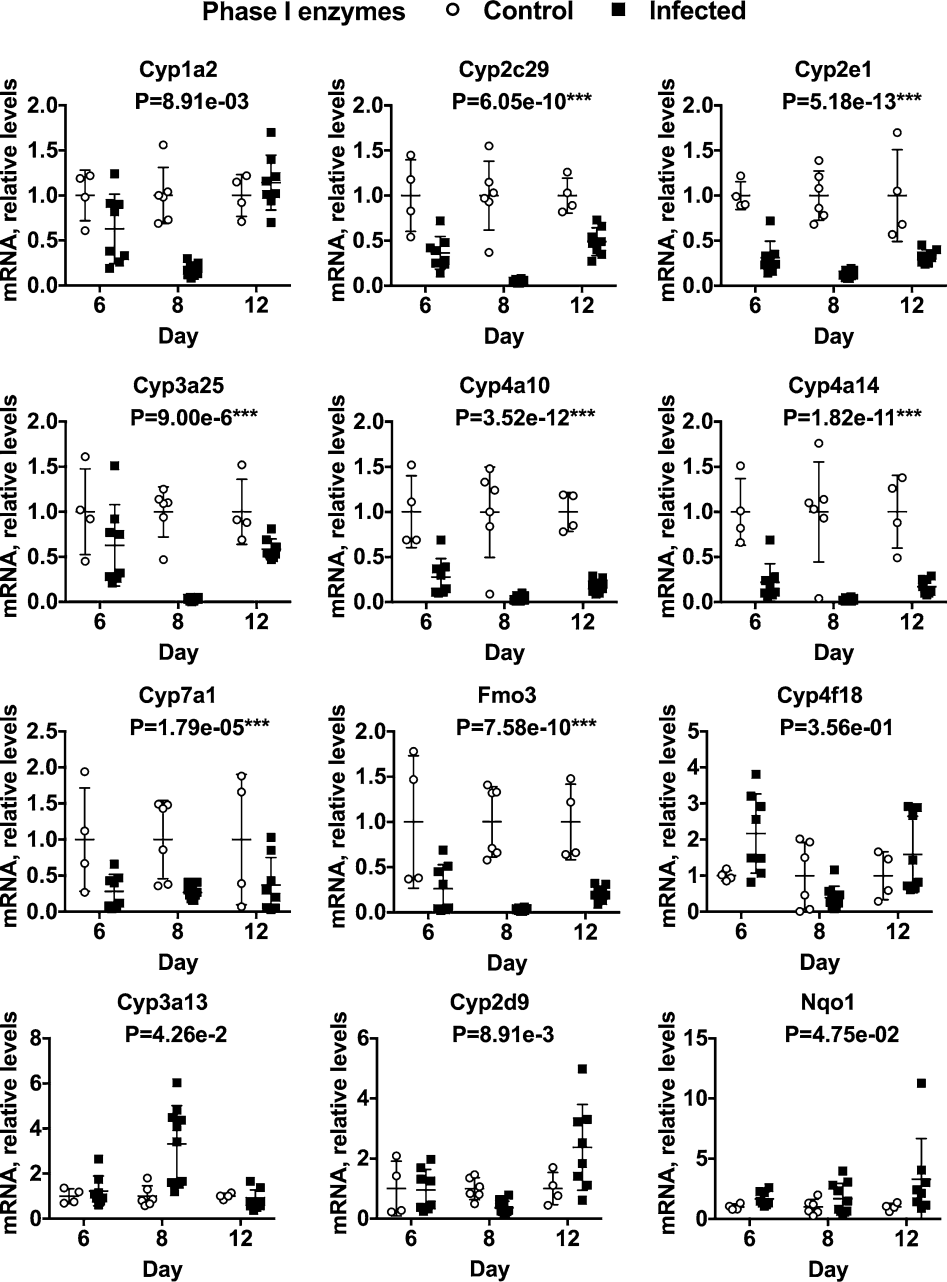
Effects of *P. chabaudi* infection on mRNA expression of phase 1 DMEs in mouse livers. Mice were euthanized at 6, 8 and 12 days after infection with *P. chabaudi* and their livers analysed by RT-qPCR for mRNA expression of phase 1 DME genes. The data are the average of two separate experiments. Values are mean ± SD, and expression in naive mice was set at 1. Differences in mRNA expression between control and infected mice among the groups were analysed by MANOVA with Bonferroni corrected one-way ANOVAs as a post hoc test. *P < 1.47e−03, **P < 7.35e−04, ***P < 1.47e−05, significantly different from naïve group. NT, not tested for significance. For days 6, 8 and 12, n = 4, 6 and 4 in the naïve groups and n = 8, 10 and 8 in the infected groups, respectively

**Fig. 3:**
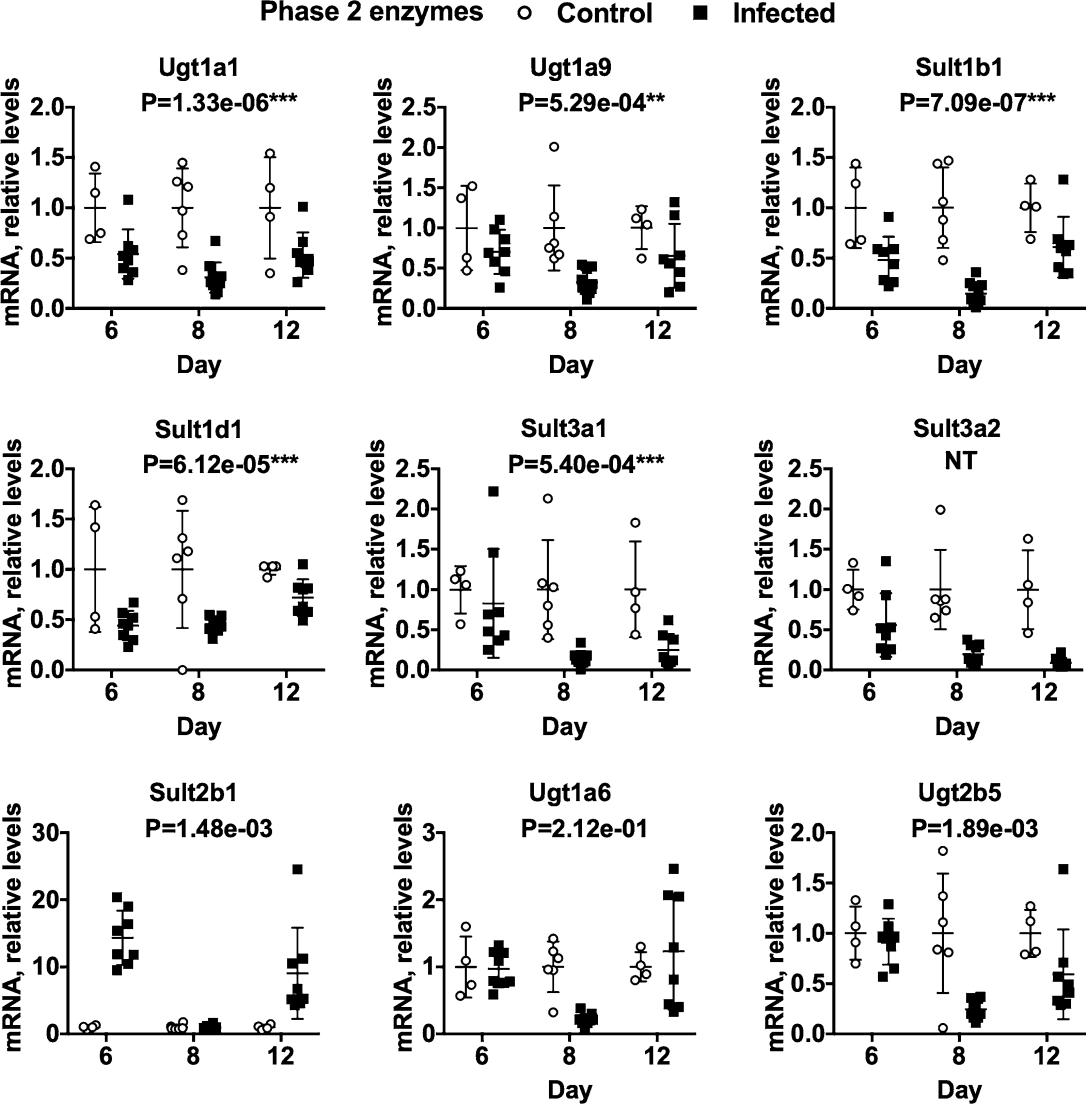
Effects of *P. chabaudi* infection on mRNA expression of phase 2 DMEs in mouse livers. Mice were euthanized at 6, 8 and 12 days after infection with *P. chabaudi* and their livers analysed by RT-qPCR for mRNA expression of phase 2 DME genes. The data are the average of two separate experiments. Values are mean ± SD, and expression in naive mice was set at 1. Differences in mRNA expression between control and infected mice among the groups were analysed by MANOVA with Bonferroni corrected one-way ANOVAs as a post hoc test. *P < 1.47e−03, **P < 7.35e−04, ***P < 1.47e−05, significantly different from naïve group. NT, not tested for significance. For days 6, 8 and 12, n = 4, 6 and 4 in the naïve groups and n = 8, 10 and 8 in the infected groups, respectively

**Fig. 4:**
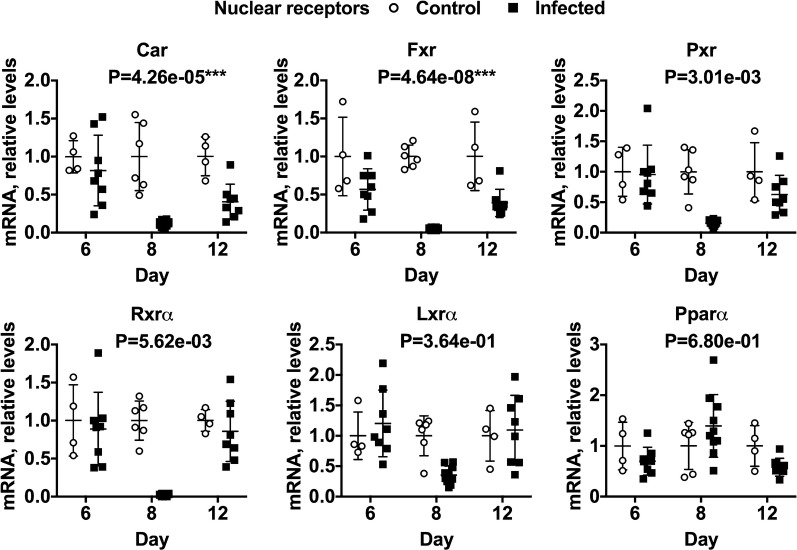
Effects of *P. chabaudi* infection on mRNA expression of nuclear receptors in mouse livers. Mice were euthanized at 6, 8 and 12 days after infection with *P. chabaudi* and their livers analysed by RT-qPCR for mRNA expression of NR genes. The data are the average of two separate experiments. Values are mean ± SD, and expression in naive mice was set at 1. Differences in mRNA expression between control and infected mice among the groups were analysed by MANOVA with Bonferroni corrected one-way ANOVAs as a post hoc test. *P < 1.47e-03, **P < 7.35e−04, ***P < 1.47e−05, significantly different from naïve group. NT, not tested for significance. For days 6, 8 and 12, n = 4, 6 and 4 in the naïve groups and n = 8, 10 and 8 in the infected groups, respectively

**Fig. 5:**
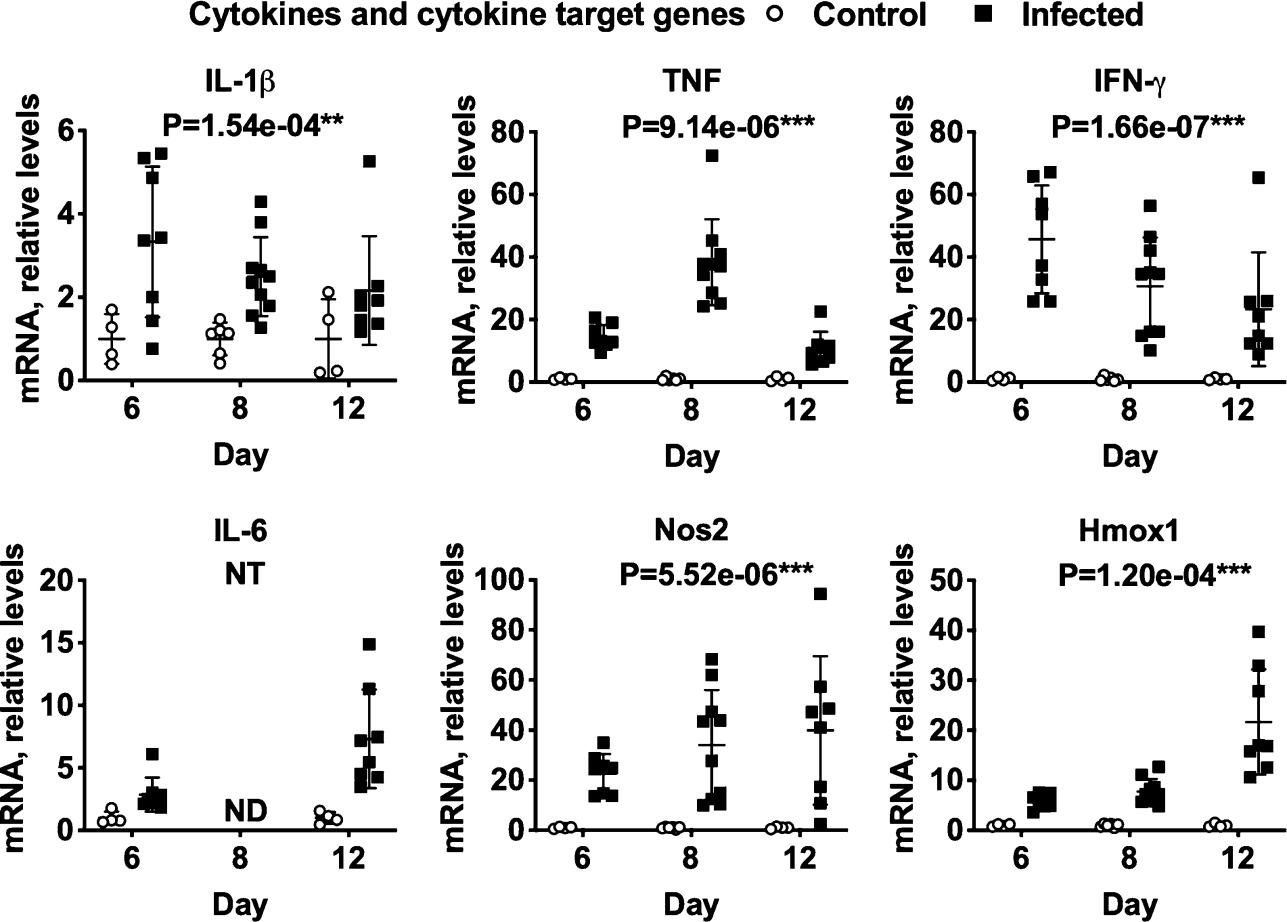
Effects of *P. chabaudi* infection on expression of cytokines and cytokine target genes in mouse livers. Mice were euthanized at 6, 8 and 12 days after infection with *P. chabaudi* and their livers analysed by RT-qPCR for mRNA expression of the analysed genes. The data are the average of two separate experiments. Values are mean ± SD, and expression in naive mice was set at 1. Differences in mRNA expression between control and infected mice among the groups were analysed by MANOVA with Bonferroni corrected one-way ANOVAs as a post hoc test. *P < 1.47e−03, **P < 7.35e−04, ***P < 1.47e−05, significantly different from naïve group. NT, not tested for significance. For days 6, 8 and 12, n = 4, 6 and 4 in the naïve groups and n = 8, 10 and 8 in the infected groups, respectively

The effects of *P. chabaudi AS* infection over time on hepatic expression of DME, nuclear receptor and inflammation-associated mRNAs are shown in Figs. 2, 3, 4, 5. Supporting data are in Additional file 5. Because a large number of DME and inflammation-associated genes were surveyed, multivariate analysis was used for the results. However, the R MANOVA function only allowed the analysis of 34 dependent variables, such that some mRNAs could not be analysed for statistical significance: this is denoted by NT (not tested) below the gene names in the figures. The P values for each analysis are also shown under the gene name. Using the Bonferroni correction for 34 samples, P values < 1.47e−03 are significant at the P < 0.05 level. mRNAs that showed no significant effect are shown in Additional file 6: Fig. S2.

The data for phase 1 and phase 2 DMEs are presented in Figs. 2 and 3, respectively. Seven of 12 P450 mRNAs analysed by MANOVA (Cyp 1a2, 2c29, 2e1, 3a25, 4a10, 4a14 and 7a1) were significantly downregulated during infection as was flavin containing monooxygenase 3 (Fmo3) (Fig. 2). There was also a trend towards down-regulation of Cyp3a11 at 6 and 12 days post-infection (Additional file 6: Fig. S2). Five of 8 Phase 2 enzyme mRNAs analysed by MANOVA were also down-regulated (Uridine 5′-diphospho-glucuronosyltransferase (Ugt) 1a1 and 1a9; sulfotransferase (Sult) 1b1, 1d1 and 3a1), and Sult3a2 showed a similar trend (Fig. 3).

The great majority of DMEs studied were profoundly down-regulated (by 50–90%) at day 8, near the peak of parasitaemia (Figs. 2, 3). However, different patterns of regulation can be discerned. Thus, Cyp2c9, Cyp2e1, Cyp4a10, Cyp4a14, Cyp7a1, Fmo3, Ugt1a1, and Sult1b1 were down-regulated at all three time points. On the other hand, Cyp1a2, Cyp3a25, Ugt1a6 and Ugt1a9, although down-regulated at 8 days post-infection, showed minimal effects at 6 and 12 days post-infection. Sult3a1 and 3a2 were little affected at 6 days, but they were down-regulated at 8 and 12 days post-infection. Conversely, Sult1d1 was down-regulated already by 6 days post-infection, but returned toward control at 12 days (Fig. 3).

There were trends towards up-regulation for 4 DME mRNAs: Cyp3a13, Cyp2d9, NAD(P)H:quinone oxidoreductase 1 (Nqo1) and Sult 2b1 (Figs. 2, 3). The most striking effect here was a biphasic increase in Sult2b1 expression that just missed the criterion for significance, with tenfold increases at days 6 and 12 and no effect on day 8 (Fig. 3).

The mRNA levels of 6 nuclear receptors involved in regulation of various DMEs were also measured (Fig. 4). Only constitutive androstane receptor (Car) and farnesoid x receptor (Fxr) were significantly affected, being down-regulated at 8 days and beginning to recover at 12 days. Pregnane x receptor (Pxr), retinoid x receptor-α (Rxrα) and liver x receptor-α (Lxrα) trended toward down-regulation at 8 days only. Peroxisome proliferator receptor-α (Pparα) was unaffected by infection.

### Cytokine and acute phase protein mRNAs

Since inflammatory responses to iRBCs sequestered in the liver sinusoids have been documented [29] and inflammation is known to modulate the expression of DMEs [30], the transcriptional profile of cytokines and key acute phase proteins in *P. chabaudi*-infected mice was determined. Expression of interleukin-1β (IL-1β), tumour necrosis factor (TNF) and interferon-γ (IFN-γ) mRNAs were all upregulated in the livers of infected mice, with TNF and IFN-γ showing the greatest increase (Fig. 5). IL-1β and IFN-γ were most affected on day 6, with subsequent declines at 8 and 12 days. TNF showed peak induction at 8 days. IL-6 was the only cytokine still elevated at 12 days post-infection (Fig. 5). These data demonstrate that *P. chabaudi* is responsible for significant hepatic inflammation.

Acute phase proteins are produced by the liver in response to signalling via receptors for inflammatory cytokines, the major mouse acute phase protein being serum amyloid A (Saa). In agreement with the transcriptional upregulation of inflammatory cytokines observed in the livers of *P. chabaudi*-infected mice, there was a > 30-fold induction of Saa1/2 mRNA at days 6 and 12 post-infection (Additional file 6: Fig. S2). Significant effects of infection were not detected on the other three acute phase protein mRNAs tested (Serum amyloid P-component (Sap), haptoglobin (Hapt), α-fibrinogen (Fga), Additional file 6: Fig. S2), although both Sap and Hapt trended toward an increase in infected mice at 4 and 12 days. Nitric oxide synthase-2 (Nos2) and heme oxygenase-1 (Hmox1) mRNAs were each strongly upregulated on days 8 and 12 (Fig. 5) suggesting that the downstream effects of *P. chabaudi* infection also include oxidative stress in the liver.

### Lxr target genes

Sult2b1, which was greatly upregulated at days 6 and 12 of infection (Fig. 3), sulfates oxysterols [31] and its increased expression is associated with reduced cellular sterol levels and reduced activity of Lxr target genes in different contexts [31–34]. Therefore, the expression of Lxr targets was measured in the samples. Of these, ATP binding cassette (Abc) subfamily G member 1 (Abcg1) was induced during *Pcc*AS infection and only Apoprotein E (Apoe) was down-regulated whereas Abc subfamily A member 1 (Abca1) and sterol regulatory element binding protein-1c (Srbp1c) were unaffected (Additional file 7: Fig. S3).

### Cytochrome P450 and NOS2 proteins

**Fig. 6:**
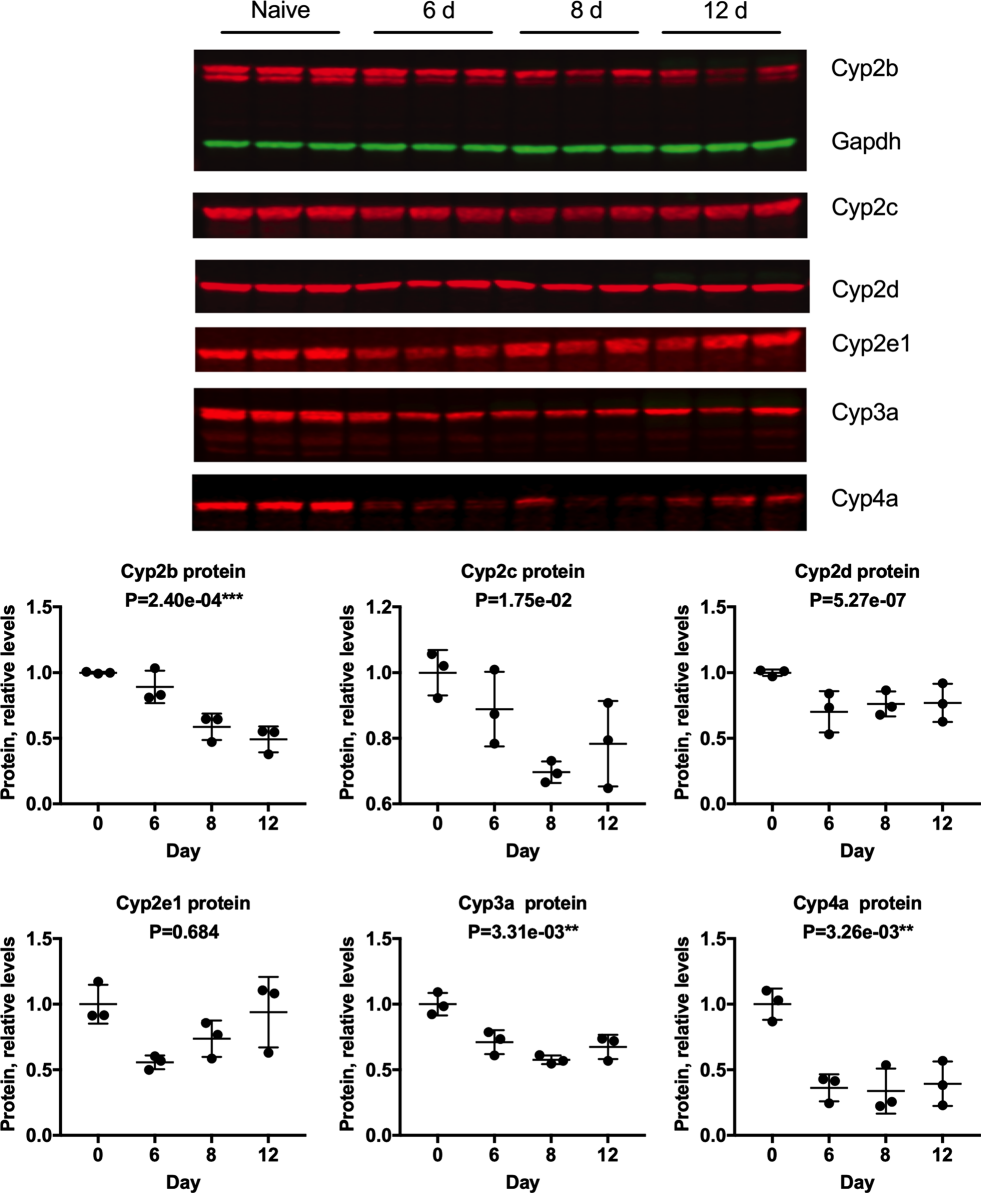
Effects of *P. chabaudi* infection on P450 protein levels in mouse livers. A new experiment was conducted, in which mice were euthanized 6, 8 and 12 days after infection with *P. chabaudi* and their livers analysed by Western blotting with IR dye detection. Values are mean ± SD, and expression in naive mice (day 0) was set at 1. The upper panel shows the Western blot images, with the Gapdh signal only shown for Cyp2b to conserve space. The lower panel shows the quantitation of these signals, normalized to Gapdh. Differences in mRNA expression between control and infected mice among the groups were analysed by MANOVA with Bonferroni corrected one-way ANOVAs as a post hoc test. *P < 7.14e−03, **P < 3.57e−03, ***P < 7.14e−04, significantly different from naïve group, n = 3 for all groups

**Fig. 7:**
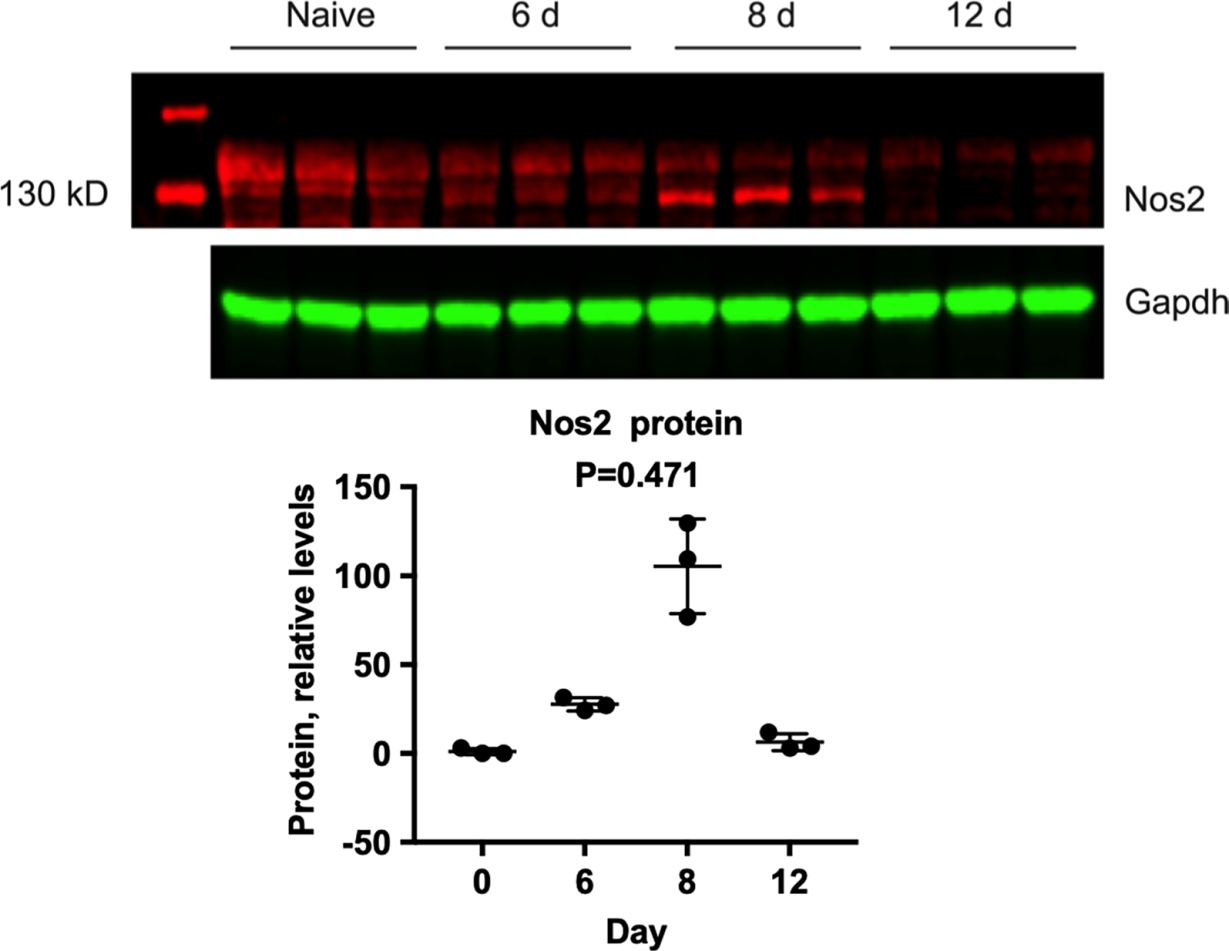
Effects of *P. chabaudi* infection on hepatic Nos2 protein levels. Samples from the same experiment in Fig. 6 were analysed by Western blotting for determination of relative Nos2 protein levels. The upper panel shows the Western blot images, and the lower panel shows the quantitation of these signals, normalized to Gapdh. Values are mean ± SD. Differences in mRNA expression between control and infected mice among the groups were analysed by MANOVA with Bonferroni correction and found not to be significant at P < 7.14e−03, n = 3 for all groups

To determine if observed changes in P450 and Nos2 mRNA expression are reflected in altered protein levels in the liver, we conducted a separate experiment and used Western blot analysis. With the exception of Cyp2e1, the antibodies used are not specific for any one mouse P450, nor are their specificities for each subfamily rigorously documented, so results should be interpreted with this caveat. Of the six subfamilies measured, Cyp2b, Cyp 3a and Cyp4a proteins were each significantly down-regulated (Fig. 6, Additional file 8), while Cyp2c, Cyp2d and Cyp2e1 proteins followed the same trend towards down-regulation. Consistent with the observations for most P450 mRNAs, the nadir was at or close to the peak of infection at day 8 in each case. For Cyp2b, Cyp2d, Cyp3a and Cyp4a proteins, levels were still below those of control (naïve) animals on day 12 post-infection (Fig. 6). Nos2 protein levels were not significantly affected although mean levels were increased on days 6 and 8 post-infection (Fig. 7; Additional file 8).

### Cassette dosing pharmacokinetic study

**Fig. 8:**
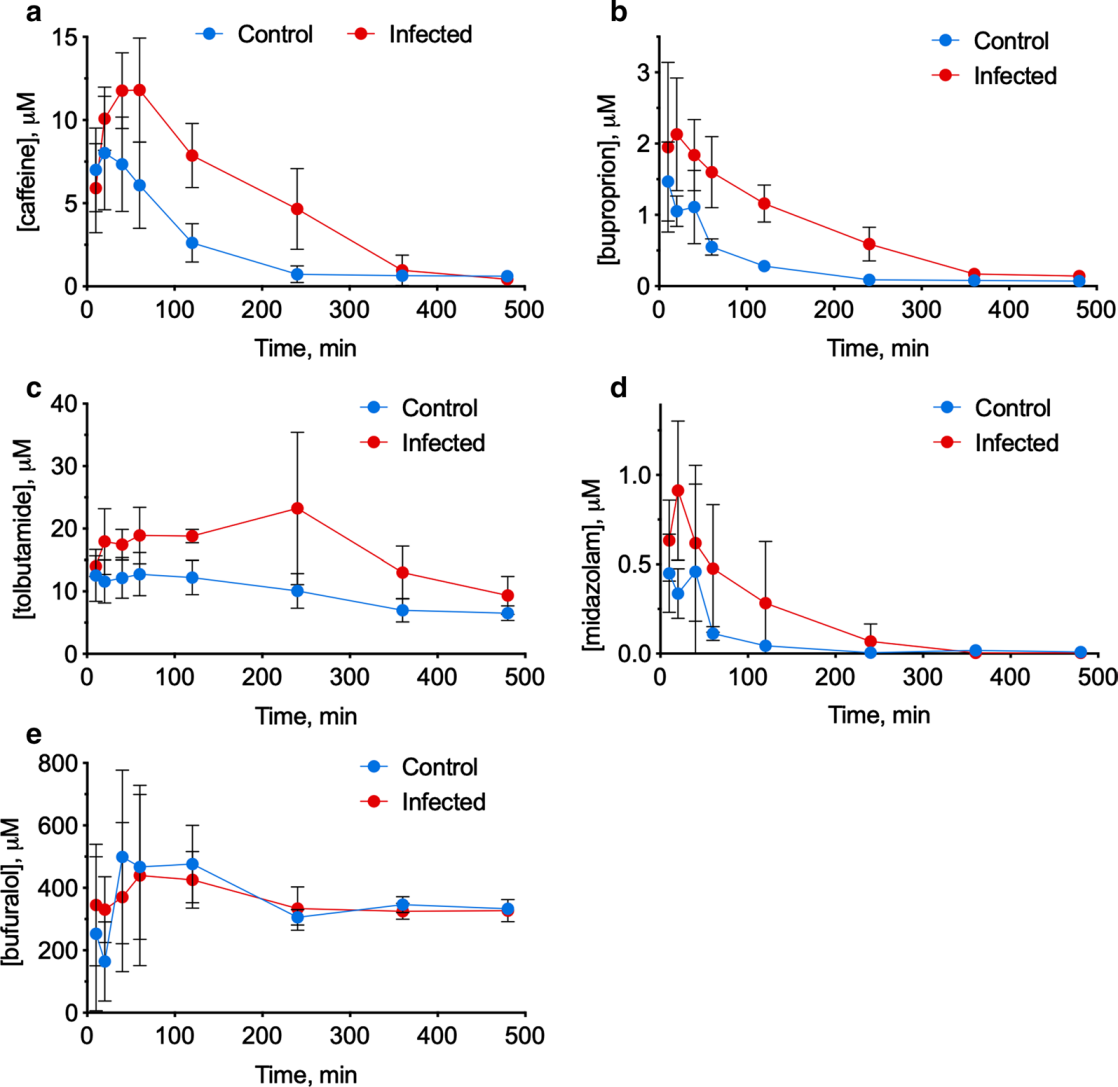
Pharmacokinetic profiles in naïve and infected mice after cassette drug dosing. 8 days after *P. chabaudi* infection, 5 naïve and 5 infected mice were administered a cocktail of 5 drugs and blood was collected at the indicated times. Drug concentrations in the blood were measured by HILIC/MS. The plots show the mean blood concentrations ± SD in each group at each time point. **a** Caffeine; **b** bupropion; **c** tolbutamide; **d** midazolam; **e** bufuralol

To determine the impact of *P. chabaudi* AS infection on DME function, the pharmacokinetics of five prototypical human P450 drug substrates were analysed after oral cassette dosing: caffeine (CYP1A2), bupropion (CYP2B6), tolbutamide (CYP2C9), midazolam (CYP3A) and bufuralol (CYP2D9) on day 8 post-infection. Parent drug concentrations were measured using a metabolomic HRMS platform consisting of HILIC coupled to a Q-Exactive HF hybrid quadropole-Orbitrap mass spectrometer, with parallel reaction monitoring. The results are shown in Fig. 8. AUC and clearance values calculated from the data are shown in Table 1, standard curves are shown in Additional file 3: Fig. S1 and supporting data are in Additional file 9. Values for bufuralol could not be calculated, but for the other four drugs, 2.7- to 4.8-fold increases in area under the curve (AUC) were observed, together with 60–70% decreases in drug clearance (Table 1).

**Table 1:** Pharmacokinetic analysis of *P. chabaudi* AS-infected mice

Drug	AUC		Clearance	
	Naïve	*Pcc*AS	Naïve	*Pcc*AS
	mM h	mM h	L/h	L/h
Caffeine	1.11 ± 0.40	2.97 ± 0.95	109 ± 68	36 ± 11
Bupropion	0.112 ± 0.26	0.391 ± 0.127	934 ± 212	280 ± 98***
Tolbutamide	10.0 ± 1.9	48.5 ± 54.2	10.3 ± 2.1	4.2 ± 2.9*
Midazolam	33.6 ± 26.9	97.9 ± 76.0	4.04 ± 1.77	1.38 ± 0.61

*P < 0.05, ***P < 0.001 compared to naïve animals using MANOVA and post hoc analysis using a general linear model with Bonferroni correction. AUC values were not tested for significance

Of the changes in clearance, those for tolbutamide (adjusted P = 0.0198) and buproprion (adjusted P = 0.00096) were statistically significant, and that for midazolam marginally failed to reach significance (adjusted P = 0.0524). Clearance of caffeine showed no statistically significant difference (adjusted P = 0.19) although its mean values showed the same trend as the other drugs. These data suggest that the downregulation in the transcriptomes of the CYP family of molecules in the livers of *P. chabaudi*-infected animals reflects a functional impairment in drug metabolism during *P. chabaudi* malaria.

## Discussion

The regulation of DMEs will dictate how well individuals respond to anti-malarial chemotherapy, and the incidence of adverse effects, by altering the levels of active drug in the system. In this study, the great majority of DME mRNAs were down-regulated more than 50% at the peak of parasitaemia in the *P. chabaudi* AS model of non-lethal malaria in mice. This downregulation is best highlighted with the observed down regulation of Cyp3a25, Cyp4a10, Cyp4a14, Cyp2e1, Fmo3 and Sutl1a1/2 mRNAs, each of which was expressed at less than 5% of that observed in naïve control mice. These effects cover the three major DME families: P450s, Ugts, and Sults. Furthermore, levels of P450 proteins of the Cyp2b, 3a and 4a subfamilies were reduced by 20–60%, with similar trends for Cyp2c, 2d and 2e1. These changes were reflected in 60–70% decreases in clearance of four P450 drug substrates. The observed changes in drug clearance are comparable in magnitude to changes in clearance of quinine (a CYP3A substrate) [4–6] and caffeine (a CYP1A2 substrate) [9, 10] reported in *Plasmodium*-infected humans suggesting that *Pcc*AS infection in mice may be a good model for determining the mechanisms behind this observation.

The only other published report of the effects of *P. chabaudi* infection on DMEs is that of De-Oliveira et al. [17] who compared the effects of *P. chabaudi* infection on hepatic microsomal P450-dependent activities in two different strains of mice. Significant down-regulation of ethoxyresorufin and benzoxyresorufin *O*-dealkylase activities were observed in C57BL/6, but not DBA mice at the peak of parasitaemia, whereas an increase in coumarin hydroxylase activity was only seen in DBA mice [17]. Other previous studies on the effects of malaria on DME mRNAs and/or proteins in rodents have been conducted in the lethal *P. berghei* ANKA model and have been limited to only a few gene products. The magnitudes of the effects observed in this study on *P. chabaudi* infection are similar to those reported for Cyp3a11, Cyp1a2 and Cyp2e1 mRNAs in *P. berghei*-infected mice [18, 20] suggesting that, despite the differences in lethality, the downregulation of these DMEs is a common feature of *Plasmodium* infections in mice.

Nitric oxide formed consequent to induction of Nos2 has been demonstrated to cause down-regulation of P450 enzyme levels and activity under various inflammatory conditions [35]. Hepatic Nos2 mRNA levels were significantly elevated in *P. chabaudi*-infected mice, especially at day 8, and Nos2 protein levels also trended upwards although not significant. The extent to which NO may contribute to the observed changes in DME protein levels during *P. chabaudi* AS infection remains to be determined.

Consistent with other disease models a small number of DMEs tended to be induced rather than suppressed in *P. chabaudi* AS-infected mice. Although the mechanisms controlling this induction are not known, hepatic Cyp3a13 was also observed to be upregulated in the dextran sodium sulfate model of inflammatory bowel disease and in the bacterial lipopolysaccharide model of sepsis [36]. Cyp2d9 is also upregulated in lipopolysaccharide treated mice [36], as well as in mice infected with the intestinal bacterial pathogen *Citrobacter rodentium* [37].

Interestingly, DMEs could be categorized based on profiling the time course of their transcription. These different transcriptional time courses suggest that there are multiple mechanisms of regulation of the enzymes during *Plasmodium* infection. However, some of the differences in time course could reflect differences in mRNA stability between transcripts for different genes. The fact that most enzymes reach their nadirs at the peak of parasitaemia (8 days, Fig. 1a) rather than correlating with liver burden (steadily increasing from 6 to 12 days, Fig. 1b) suggests that hepatic sequestration of iRBCs is not the main factor regulating DME expression. On the other hand, the down-regulation of Sult3a1 and Sult3a2 does correlate with liver burden, which may suggest such a mechanism for these enzymes.

In other models of inflammation and infection, inflammatory cytokines such as IL-1β, TNF, IL-6 and IFN-γ are thought to be the major mediators of DME down-regulation [38]. Interestingly, the patterns of IL-1β and IFN-γ induction in the liver (Fig. 4b) correlated best with e.g. Sult1d1, which was downregulated at day 6 and trended back towards control levels at day 12. TNF expression peaked at 8 days, correlating (negatively) with the majority of DMEs measured. Since these are cytokine mRNAs measured in the liver, and not cytokine proteins, caution should be exercised in drawing any mechanistic inferences, but these data merit further investigation.

Nuclear receptors that positively regulate DME genes are down-regulated in other models of inflammatory diseases such as the administration of the toll-like receptor agonists bacterial lipopolysaccharide [39] and lipotechoic acid [40] and modulation appears to be in part regulated by IL-6 [41]. In some cases nuclear receptor down-regulation seems to be mechanistically involved in the observed down-regulation of the DMEs [40–42]. Indeed, there is good evidence that down-regulation of retinoid X receptor-α (Rxrα), the heterodimerization partner for several other nuclear receptors, plays a central role in the down-regulation of DMEs by IL-6 in human hepatocytes [43]. In *P. chabaudi*-infected mice, Fxr was downregulated at all three time points measured when compared to naïve control mice. Car was also down-regulated, but only at 8 days post-infection and Rxrα, Pxr and Lxrα followed the same trend. Rxrα suppression was dramatic with expression levels less than 1% of that measured in naïve control animals. While this might contribute to DME down-regulation at 8 and 12 days, it cannot explain the down-regulation of transcription of some DMEs at 6 days post infection.

The data shows that expression of Lxrα trended towards downregulation (Fig. 4). This was coupled with a > 10-fold, biphasic upregulation of Sult2b1 (Fig. 3). Among other functions, Sult2b1 sulfates oxysterols [31] and as noted above, reduced cellular oxysterol levels caused by increased expression of Sult2b1 have been associated with reduced expression of Lxr target genes. The observations of reduced expression of the Lxr target Cyp7a1 [44] (Fig. 2) and Apoe [45] (Additional file 5) are consistent with this idea and also with the observation that Lxrα itself was down-regulated. However, another Lxr target gene, Abcg1 [45] was upregulated more than fivefold, whilst transcription of both Srbp1c [31] and Abca1 [46] were comparatively unaffected by infection, suggesting that there may be redundancy in the Lxr pathway involved in regulation of these genes during *Plasmodium* infection. The upregulation of Sult2b1 is a novel observation that suggests a possible role of this enzyme in the host response to *Plasmodium* infection. More work will be needed to determine if Lxrα is activated or inactivated in this model of malaria and if this is related to Sult2b1 upregulation and enhanced sulfation of oxysterols.

The great majority of anti-malarial drugs are eliminated via metabolic pathways, and principally by cytochrome P450 enzymes [8, 47–60] (Table 2). The down-regulation of a broad swathe of enzymes in the *P. chabaudi* mouse model suggests that the therapeutic efficacy of drugs that require metabolism to be effective, such as primaquine, tafenoquine and proguanil could be compromised at stages of the disease when down-regulation is at its peak; the different times courses for different enzymes and relatively high interindividual variability found even in inbred mice suggest that this will be highly variable in people. Conversely, drugs (and metabolites) that are inactivated by metabolism such as dihydroartemisinin, mefloquine, quinine and lumefantrine can be expected to accumulate to higher levels during these periods, potentially resulting in increased side effects and toxicity. It must also be emphasized that these findings have potential consequences for individuals with untreated, uncomplicated malaria who take medications for symptoms or for co-existing conditions.

**Table 2:** Important enzymes in the metabolism of anti-malarial drugs

Drug	Metabolism outcome	Enzymes involved	References
Artemisinin	Inactivation	CYP2B6, 3A4, 2D6	[47, 48]
Dihydroartemisinin (DHA)	Inactivation	UGT1A9, 2B7	[49]
Artesunate	DHA formation	CYP2A6	[47]
Arteether	DHA formation	CYP3A4>2B6,3A5	[50]
Artemether	DHA formation	CYP3A4	[51]
Quinine	Inactivation	CYP3A4	[8, 47]
Mefloquine	Inactivation	CYP3A4	[52]
Chloroquine	Active metabolite	CYP2C8, 3A4>2D6	[53]
Piperaquine	Active metabolite	CYP3A4	[54]
Primaquine	Required for activity	CYP2D6	[55]
Tafenoquine	Required for activity	CYP2D6	[59]
Lumefantrine	Inactivation	CYP3A4	[51]
Amodiaquine	Reduced activity	CYP2C8	[56]
Proguanil	Required for activity	CYP2C19	[57]
Sulfadoxine	Inactivation	NAT2	[58]
Clindamycin	Inactivation	CYP3A4	[60]

While the P450 substrates employed in the pharmacokinetic experiment (Fig. 8) are used for in vivo P450 phenotyping in humans, they have not been rigorously evaluated in mice. Nevertheless, it is reasonable to attribute the substantial decreases in clearance of four different drugs to the observed down-regulation of most P450 mRNAs and proteins studied, although changes to hepatic blood flow and plasma protein binding could also contribute to the altered drug clearance in this *Plasmodium* infection model. In humans with uncomplicated malaria, there was a small increase in plasma protein binding of quinine in the plasma in one study [61], whereas another found no effect [6]. In the latter study, liver blood flow measured with iodocyanine green was reported to be reduced by 40% compared to convalescing individuals. If such changes in blood flow also occur in mice, the relative contribution of reduced liver blood flow to the changes in clearance would be expected to be greatest for high-clearance substrates, such as bupropion.

This study used Orbitrap-based high-resolution mass metabolomics MS platform to perform determination of clearance for multiple substrates after cassette dosing. Among the advantages of this approach are that full-scan HRMS data allows for the metabolomic signatures of the experimental condition (in this case, malaria) to be yielded in the same sample run, and analysed later. It may be possible from such data to correlate levels of endogenous P450 substrates and other biomarkers with the measured drug pharmacokinetics, and there is no reason why this approach could not also be taken in human subjects. Four of the five drugs studied provided reliable data, with measurable pharmacokinetic parameters that demonstrated clear differences between naïve and infected mice. The atypical plasma profile of bufuralol may be the result of an analytical artifact caused by the co-elution of an in-source fragment from a bufuralol metabolite, likely hydroxybufuralol. This is the likeliest explanation for high background observed for bufuralol M+H in all samples analysed from mice that received the drug cocktail, but not in mice that did not receive the drug cocktail.

## Conclusions

In summary, the data demonstrate that most drug metabolizing enzymes are significantly affected in a rodent model of non-lethal malaria, and this leads to changes in drug clearance of sufficient magnitude that could be clinically significant at the same magnitude in humans with malaria. This data paves the way for undertaking further studies in humans with uncomplicated malaria.

## Additional files

**Additional file 1: Table S1.** Primers used for qPCR.

**Additional file 2: Table S2.** Antibodies used for Western blotting.

**Additional file 3: Fig. S1.** Standard curves for caffeine, buproprion, tolbutamide, midazolam and bufuralol measured by HILIC/MS.

**Additional file 4.** PccAS burdens data.

**Additional file 5.** mRNA supporting data.

**Additional file 6: Fig. S2. ** Additional analyses of mRNA expression in mouse livers.

**Additional file 7: Fig. S3.** Effects of *P. chabaudi* AS infection on mRNA expression of LXR target genes in mouse livers.

**Additional file 8.** Protein/Western blot data.

**Additional file 9.** Pharmacokinetic/MS data.

## Data Availability

All data generated or analysed during this study are included in this published article and Additional files.

## Abbreviations

Agp: α1-acid glycoprotein
Apoe: apoprotein E
AUC: area under the curve
Car: constitutive androstane receptor
DME: drug metabolizing enzyme
Fga: fibrinogen A
Fmo3: flavin monooxygenase-3
Fxr: farnesoid X receptor
Gapdh: glyceraldehyde-3-phosphate dehydrogenase
Hapt: haptoglobin
HILIC: hydrophilic interaction chromatography
HRMS: high resolution mass spectrometry
Hmox1: heme oxygenase-1
IFN-ɣ: interferon-ɣ
IL-1β: interleukin-1β
IL-6: interleukin-6
iRBC: infected red blood cell
Lxrα: liver X receptor-α
MSP1: merozoite surface protein-1
Nos2: nitric oxide synthase-2
Nqo1: NAD(P)H-quinone oxidoreductase 1
P450: cytochrome P450
*Pb*ANKA: ANKA strain of *P. berghei*
*Pcc*AS: AS strain of *P. chabaudi chabaudi*
Pparα: peroxisome proliferator-activated receptor-α
Pxr: pregnane X receptor
QC: quality control
RBC: red blood cell
RT-qPCR: reverse transcriptase-real time polymerase chain reaction
Rxrα: retinoid X receptor-α
Saa: serum amyloid A
Sap: serum amyloid P
Srbp1c: sterol regulatory element binding protein-1c
Sult: sulfotransferase
TNF: tumour necrosis factor
Ugt: UDP-glucoronosyltransferase

## References

[1] WHO (2016). World malaria report 2016.

[2] Trenholme GM, Williams RL, Rieckmann KH, Frischer H, Carson PE (1976). Quinine disposition during malaria and during induced fever. Clin Pharmacol Ther.

[3] Sabchareon A, Chongsuphajaisiddhi T, Attanath P (1982). Serum quinine concentrations following the initial dose in children with falciparum malaria. Southeast Asian J Trop Med Public Health.

[4] White NJ, Looareesuwan S, Warrell DA, Warrell MJ, Bunnag D, Harinasuta T (1982). Quinine pharmacokinetics and toxicity in cerebral and uncomplicated falciparum malaria. Am J Trop Med Hyg.

[5] Supanaranond W, Davis TM, Pukrittayakamee S, Silamut K, Karbwang J, Molunto P (1991). Disposition of oral quinine in acute falciparum malaria. Eur J Clin Pharmacol.

[6] Pukrittayakamee S, Looareesuwan S, Keeratithakul D, Davis TM, Teja-Isavadharm P, Nagachinta B (1997). A study of the factors affecting the metabolic clearance of quinine in malaria. Eur J Clin Pharmacol.

[7] Kloprogge F, Jullien V, Piola P, Dhorda M, Muwanga S, Nosten F (2014). Population pharmacokinetics of quinine in pregnant women with uncomplicated *Plasmodium falciparum* malaria in Uganda. J Antimicrob Chemother.

[8] Zhang H, Coville PF, Walker RJ, Miners JO, Birkett DJ, Wanwimolruk S (1997). Evidence for involvement of human CYP3A in the 3-hydroxylation of quinine. Br J Clin Pharmacol.

[9] Wilairatana P, Looareesuwan S, Vanijanonta S, Charoenlarp P, Wittayalertpanya S (1994). Hepatic metabolism in severe falciparum malaria: caffeine clearance study. Ann Trop Med Parasitol.

[10] Akinyinka OO, Sowunmi A, Honeywell R, Renwick AG (2000). The pharmacokinetics of caffeine in Nigerian children suffering from malaria and kwashiorkor. Eur J Clin Pharmacol.

[11] Akinyinka OO, Sowunmi A, Honeywell R, Renwick AG (2000). The effects of acute falciparum malaria on the disposition of caffeine and the comparison of saliva and plasma-derived pharmacokinetic parameters in adult Nigerians. Eur J Clin Pharmacol.

[12] McCarthy JS, Furner RL, Van Dyke K, Stitzel RE (1970). Effects of malarial infection on host microsomal drug-metabolizing enzymes. Biochem Pharmacol.

[13] Kokwaro GO, Glazier AP, Ward SA, Breckenridge AM, Edwards G (1993). Effect of malaria infection and endotoxin-induced fever on phenacetin O-deethylation by rat liver microsomes. Biochem Pharmacol.

[14] Glazier AP, Kokwaro GO, Edwards G (1994). Possible isozyme-specific effects of experimental malaria infection with *Plasmodium berghei* on cytochrome P450 activity in rat liver microsomes. J Pharm Pharmacol.

[15] Pandey AV, Srivastava AK, Tekwani BL, Pandey VC (1996). Effect of *Plasmodium yoelii* infection on constitutive and phenobarbitone inducible mixed function oxidase system of mice. J Parasitic Dis.

[16] De-Oliveira AC, Da-Matta AC, Paumgartten FJ (2006). Plasmodium berghei (ANKA): infection induces CYP2A5 and 2E1 while depressing other CYP isoforms in the mouse liver. Exp Parasitol.

[17] De-Oliveira AC, Carvalho RS, Paixao FH, Tavares HS, Gueiros LS, Siqueira CM (2010). Up- and down-modulation of liver cytochrome P450 activities and associated events in two murine malaria models. Malar J.

[18] Carvalho RS, Friedrich K, De-Oliveira AC, Suarez-Kurtz G, Paumgartten FJ (2009). Malaria downmodulates mRNA expression and catalytic activities of CYP1A2, 2E1 and 3A11 in mouse liver. Eur J Pharmacol.

[19] Uhl K, Grace JM, Kocisko DA, Jennings BT, Mitchell AL, Brewer TG (1999). Effects of *Plasmodium berghei* infection on cytochromes P-450 2E1 and 3A2. Eur J Drug Metab Pharmacokinet.

[20] Cressman AM, McDonald CR, Silver K, Kain KC, Piquette-Miller M (2014). Malaria infection alters the expression of hepatobiliary and placental drug transporters in pregnant mice. Drug Metab Dispos.

[21] Stephens R, Culleton RL, Lamb TJ (2012). The contribution of *Plasmodium chabaudi* to our understanding of malaria. Trends Parasitol.

[22] Mimche PN, Brady LM, Bray CF, Lee CM, Thapa M, King TP (2015). The receptor tyrosine kinase EphB2 promotes hepatic fibrosis in mice. Hepatology.

[23] Mimche SM, Nyagode BA, Merrell MD, Lee CM, Prasanphanich NS, Cummings RD (2014). Hepatic cytochrome P450s, phase II enzymes and nuclear receptors are downregulated in a Th2 environment during *Schistosoma mansoni* infection. Drug Metab Dispos.

[24] Chaluvadi MR, Kinloch RD, Nyagode BA, Richardson TA, Raynor MJ, Sherman M (2009). Regulation of hepatic cytochrome P450 expression in mice with intestinal or systemic infections of *Citrobacter rodentium*. Drug Metab Dispos.

[25] Richardson TA, Morgan ET (2005). Hepatic cytochrome P450 gene regulation during endotoxin-induced inflammation in nuclear receptor knockout mice. J Pharmacol Exp Ther.

[26] Scheer N, McLaughlin LA, Rode A, Macleod AK, Henderson CJ, Wolf CR (2014). Deletion of 30 murine cytochrome p450 genes results in viable mice with compromised drug metabolism. Drug Metab Dispos.

[27] Go YM, Walker DI, Liang Y, Uppal K, Soltow QA, Tran V (2015). Reference standardization for mass spectrometry and high-resolution metabolomics applications to exposome research. Toxicol Sci.

[28] Livak KJ, Schmittgen TD (2001). Analysis of relative gene expression data using real-time quantitative PCR and the 2^−ΔΔCT^ method. Methods.

[29] Brugat T, Cunningham D, Sodenkamp J, Coomes S, Wilson M, Spence PJ (2014). Sequestration and histopathology in *Plasmodium chabaudi* malaria are influenced by the immune response in an organ-specific manner. Cell Microbiol.

[30] Mallick P, Taneja G, Moorthy B, Ghose R (2017). Regulation of drug-metabolizing enzymes in infectious and inflammatory disease: implications for biologics-small molecule drug interactions. Expert Opin Drug Metab Toxicol.

[31] Chen W, Chen G, Head DL, Mangelsdorf DJ, Russell DW (2007). Enzymatic reduction of oxysterols impairs LXR signaling in cultured cells and the livers of mice. Cell Metab.

[32] Bensinger SJ, Bradley MN, Joseph SB, Zelcer N, Janssen EM, Hausner MA (2008). LXR signaling couples sterol metabolism to proliferation in the acquired immune response. Cell.

[33] Bai Q, Xu L, Kakiyama G, Runge-Morris MA, Hylemon PB, Yin L (2011). Sulfation of 25-hydroxycholesterol by SULT2B1b decreases cellular lipids via the LXR/SREBP-1c signaling pathway in human aortic endothelial cells. Atherosclerosis.

[34] Lo Sasso G, Celli N, Caboni M, Murzilli S, Salvatore L, Morgano A (2010). Down-regulation of the LXR transcriptome provides the requisite cholesterol levels to proliferating hepatocytes. Hepatology.

[35] Aitken AE, Richardson TA, Morgan ET (2006). Regulation of drug-metabolizing enzymes and transporters in inflammation. Annu Rev Pharmacol Toxicol.

[36] Chaluvadi MR, Nyagode BA, Kinloch RD, Morgan ET (2009). TLR4-dependent and -independent regulation of hepatic cytochrome P450 in mice with chemically induced inflammatory bowel disease. Biochem Pharmacol.

[37] Nyagode BA, Lee CM, Morgan ET (2010). Modulation of hepatic cytochrome P450s by Citrobacter rodentium infection in interleukin-6- and interferon-{gamma}-null mice. J Pharmacol Exp Ther.

[38] Harvey RD, Morgan ET (2014). Cancer, inflammation, and therapy: effects on cytochrome p450-mediated drug metabolism and implications for novel immunotherapeutic agents. Clin Pharmacol Ther.

[39] Beigneux AP, Moser AH, Shigenaga JK, Grunfeld C, Feingold KR (2002). Reduction in cytochrome P-450 enzyme expression is associated with repression of CAR (constitutive androstane receptor) and PXR (pregnane X receptor) in mouse liver during the acute phase response. Biochem Biophys Res Commun.

[40] Shah P, Guo T, Moore DD, Ghose R (2014). Role of constitutive androstane receptor in Toll-like receptor-mediated regulation of gene expression of hepatic drug-metabolizing enzymes and transporters. Drug Metab Dispos.

[41] Teng S, Piquette-Miller M (2005). The involvement of the pregnane X receptor in hepatic gene regulation during inflammation in mice. J Pharmacol Exp Ther.

[42] Yang J, Hao C, Yang D, Shi D, Song X, Luan X (2010). Pregnane X receptor is required for interleukin-6-mediated down-regulation of cytochrome P450 3A4 in human hepatocytes. Toxicol Lett.

[43] Keller R, Klein M, Thomas M, Drager A, Metzger U, Templin MF (2016). Coordinating role of RXR alpha in downregulating hepatic detoxification during inflammation revealed by fuzzy-logic modeling. PLoS Comput Biol.

[44] Peet DJ, Turley SD, Ma W, Janowski BA, Lobaccaro JM, Hammer RE (1998). Cholesterol and bile acid metabolism are impaired in mice lacking the nuclear oxysterol receptor LXR alpha. Cell.

[45] Laffitte BA, Repa JJ, Joseph SB, Wilpitz DC, Kast HR, Mangelsdorf DJ (2001). LXRs control lipid-inducible expression of the apolipoprotein E gene in macrophages and adipocytes. Proc Natl Acad Sci USA.

[46] Repa JJ, Turley SD, Lobaccaro JA, Medina J, Li L, Lustig K (2000). Regulation of absorption and ABC1-mediated efflux of cholesterol by RXR heterodimers. Science.

[47] Li XQ, Bjorkman A, Andersson TB, Gustafsson LL, Masimirembwa CM (2003). Identification of human cytochrome P(450)s that metabolise anti-parasitic drugs and predictions of in vivo drug hepatic clearance from in vitro data. Eur J Clin Pharmacol.

[48] Svensson US, Ashton M (1999). Identification of the human cytochrome P450 enzymes involved in the in vitro metabolism of artemisinin. Brit J Clin Pharmacol.

[49] Ilett KF, Ethell BT, Maggs JL, Davis TM, Batty KT, Burchell B (2002). Glucuronidation of dihydroartemisinin in vivo and by human liver microsomes and expressed UDP-glucuronosyltransferases. Drug Metab Dispos.

[50] Grace JM, Aguilar AJ, Trotman KM, Peggins JO, Brewer TG (1998). Metabolism of beta-arteether to dihydroqinghaosu by human liver microsomes and recombinant cytochrome P450. Drug Metab Dispos.

[51] Lefevre G, Bindschedler M, Ezzet F, Schaeffer N, Meyer I, Thomsen MS (2000). Pharmacokinetic interaction trial between co-artemether and mefloquine. Eur J Pharm Sci.

[52] Fontaine F, de Sousa G, Burcham PC, Duchene P, Rahmani R (2000). Role of cytochrome P450 3A in the metabolism of mefloquine in human and animal hepatocytes. Life Sci.

[53] Projean D, Baune B, Farinotti R, Flinois JP, Beaune P, Taburet AM (2003). In vitro metabolism of chloroquine: identification of CYP2C8, CYP3A4, and CYP2D6 as the main isoforms catalyzing *N*-desethylchloroquine formation. Drug Metab Dispos.

[54] Lee TM, Huang L, Johnson MK, Lizak P, Kroetz D, Aweeka F (2012). In vitro metabolism of piperaquine is primarily mediated by CYP3A4. Xenobiotica.

[55] Pybus BS, Marcsisin SR, Jin X, Deye G, Sousa JC, Li Q (2013). The metabolism of primaquine to its active metabolite is dependent on CYP 2D6. Malar J.

[56] Li XQ, Bjorkman A, Andersson TB, Ridderstrom M, Masimirembwa CM (2002). Amodiaquine clearance and its metabolism to *N*-desethylamodiaquine is mediated by CYP2C8: a new high affinity and turnover enzyme-specific probe substrate. J Pharmacol Exp Ther.

[57] Wright JD, Helsby NA, Ward SA (1995). The role of *S*-mephenytoin hydroxylase (CYP2C19) in the metabolism of the antimalarial biguanides. Br J Clin Pharmacol.

[58] Barraviera B, Pereira PC, Machado JM, de Souza MJ, Lima CR, Curi PR (1991). Isoniazid acetylating phenotype in patients with paracoccidioidomycosis and its relationship with serum sulfadoxin levels, glucose-6-phosphate dehydrogenase and glutathione reductase activities. Rev Soc Bras Med Trop.

[59] Marcsisin SR, Sousa JC, Reichard GA, Caridha D, Zeng Q, Roncal N (2014). Tafenoquine and NPC-1161B require CYP 2D metabolism for anti-malarial activity: implications for the 8-aminoquinoline class of anti-malarial compounds. Malar J.

[60] Wynalda MA, Hutzler JM, Koets MD, Podoll T, Wienkers LC (2003). In vitro metabolism of clindamycin in human liver and intestinal microsomes. Drug Metab Dispos.

[61] Silamut K, White NJ, Looareesuwan S, Warrell DA (1985). Binding of quinine to plasma proteins in falciparum malaria. Am J Trop Med Hyg.

