# Supplementary material for: A non-lethal malarial infection results in reduced drug metabolizing enzyme expression and drug clearance in mice
Source: Malar J. 2019 Jul 12;18:234. doi: 10.1186/s12936-019-2860-5 (PMC6624958; doi:10.1186/s12936-019-2860-5)
Supplement: Supplementary file 1 — Additional file 1: Table S1. Primers used for qPCR. [file 12936_2019_2860_MOESM1_ESM.pdf]

Table S1. Primers used for qPCR

Primers without references were designed using Primer Express® software v2.0 (Applied Biosystems)

Gene	Forward primer	Reverse primer	Reference
Gapdh	AGCCAAACGGGTCATCATCTC	GGGGGCTAAGCAGTTGGTG	(1)
Cyp1a2	TGGAGCTGGCTTTGACACAG	CGTTAGGCCATGTCACAAGTAGC	(2)
Cyp2c29	AAGGTTTTGGCGTTGTTTTTAGC	AGCCTTTGGTTTTCCTCAGTTCTT	(3)
Cyp2d9	CCTCCAAGGTCAGAAGTCCTTCA	CGATTTCTTGTTGGACTCTGCG	(4)
Cyp2e1	AAGCGCTTCGGGCCAG	TAGCCATGCAGGACCACGA	(2)
Cyp3a11	GGATGAGATCGATGAGGCTCTG	CAGGTATTCCATCTCCATCACAGT	(2)
Cyp3a13	CCTCTGCCTTTCTTGGGGACGAT	CCGCCGGTTTGTGAAGGTAGAGT	(3)
Cyp3a25	TTGGAGGCCTGAACTGCTAAAG	CTGTGGGCCCTCATAAAACC	(1)
Cyp4a10	TGTCCCAGGCATTGTCAGAGA	CCTTCGGGTTGTGGTGGAGA	(3)
Cyp4a14	CCCAAAGGTATCACAGCCACAA	CAGCAATTCAAAGCGGAGCAG	(3)
Cyp4f18	AGAGCCTGGTGCGAACCTT	TGGAATATGCGGATGACTGG	(5)
Cyp7a1	TGTCTGCGAGGGCTGGAGCA	CCAGCCTGGGATGCTATGGGC	(6)
Fmo3	CAGCATTTACCAATCGGTCTTC	TGACTTCCCATTTGCCAGTAG	(7)
Sult1b1	TTGAAGAGTTCCAGAGCA	GAACCTTTGGAGGTGATAACA	(8)
Sult1d1	GAGGGAGTTAGTGGATGTT	TCACTGACCCAAGTTGTTC	(8)
Sult2b1	CAGTCTGGCTGAGAACACTA	TTCAGGATTAAGCAGACGAT	(8)
Sult3a1	CATCGGAACAGAACTGAAAACA	TATGGAATGTGGGAACTGAAGA	(9)
Sult3a2	GATATGGATGCTGTTGTGAGACAA	TCCAGTCTCCAACGATACCTTTG	(9)
Ugt1a1	CCAGCAGAAGGGGCACGAAGTTG	TGACCACGCGCAGCAGAAAAGAAT	(10)
Ugt1a2	TGCCCCTCCGAGGAATCTCAG	CTCCCGCACAACATCCCTCATG	(10)
Ugt1a6	CTGGCTGATGGTGGCTGACTG	ACTGAGGCCCAAAGCACTAGGAA	(10)
Ugt1a9	CAGAGGGCATGAGGTTGTGGTA	TCCTGCAGTGTGAAAATGTTAGTT	(10)
Ugt2b5	ACCCCAGCAACTTTAGGACACAAT	TGATAGATCGCCTCGTAGACACCA	(10)
Car	CAGACCCGGAGTTACCCAAAGAGA	GAGCAGCGGCGTCATAGCAGA	(1)
Fxr	ACGGGGGCAACTGCGTGATG	CGCCCTTCGCTGTCGTCCTC	(1)
Lxra	CTCAATGCCTGATGTTTCTCCT	TCCAACCCTATCCCTAAAGCAA	(11)
Ppara	GGGGGTGGGGAGCATTTTCA	GGAGGGGCCTAGAGGAGCATTTAC	(1)
Pxr	CGCGGAGAAGACGGCAGCATC	CCCAGGTTCCCGTTTCCGTGTC	(1)
Rxra	GGCGCCATCTTTGACAGGGTGCTA	CCGGCTGCTCAGGGTACTTGTGTT	(1)
Nos2	CTTCACGGGTCAGAGCCACAGTCC	GAGCCAAAGCCAAACACAGCATAC	(1)
Hmox1	AGGAGCTGCACCGAAGGGCT	GTGGCTGGCGTGCAAGGGAT	
Abca1	GGTTTGGAGATGGTTATACAATAGT TGT	TTCCCGGAAACGCAAGTC	
Srbp1c	GATGTGCGAACTGGACACAG	CATAGGGGGCGTCAAACAG	(12)
Abcg1	AGTCTGACGATGACCAGTGTTGAG	CTTCCTAGTTAGAAAAGGAAACGAA CAGT	
Apoe	GCCGTGCTGTTGGTCACA	TGCCACTCGAGCTGATCTGT	
IL1b	CAACCAACAAGTGATATTCTCCATG	GATCCACACTCTCCAGCTGCA	(13)
IL6	CAACCACGGCCTTCCCTACT	TCATTTCCACGATTTCCCAGAG	(3)
Ifng	AACGCTACACACTGCATCTTGG	GCCGTGGCAGTAACAGCC	(14)
Tnfa	GACAAGGCTGCCCCGACTACG	CTTGGGGCAGGGGCTCTTGAC	(3)
Agp	CTTCGGGAGTCTCAAACAATAGGT	GACAGTCCCCGTTTCTTCTCATCC	(3)
Fga	AGCAGCCCAGCCACAAGAAAAGAG	CGTCGAGACTGCCGCTAAAACTGT	(3)
Hapt	TGAACACAGTCGCTGGAGAG	GCTGCCTTTGGCATCCATAG	(15)
Saa1/2	GTAATTGGGGTCTTTGCC	TTCTGCTCCCTGCTCCTG	(16)
Sap	AGGGCAGAGACAATGAGCTACT	GGACTCCCAAGTGGTACATAGG	(17)
*Pcc*AS MSP1	ACAGTAACACAAGAAGGAAC	GATACTTGTGTTGATGCTGG	(18)

References

1. Mimche SM, Nyagode BA, Merrell MD, Lee CM, Prasanphanich NS, Cummings RD, et al. Hepatic cytochrome P450s, phase II enzymes and nuclear receptors are downregulated in a Th2 environment during Schistosoma mansoni infection. Drug Metab Dispos. 2014;42:134-40.
2. Pan J, Xiang Q, Ball S. Use of a novel real-time quantitative reverse transcription-polymerase chain reaction method to study the effects of cytokines on cytochrome P450 mRNA expression in mouse liver. Drug Metab Dispos. 2000;28:709-13.
3. Richardson TA, Morgan ET. Hepatic cytochrome P450 gene regulation during endotoxin-induced inflammation in nuclear receptor knockout mice. J Pharmacol Exp Ther. 2005;314:703-9.
4. Blume N, Leonard J, Xu ZJ, Watanabe O, Remotti H, Fishman J. Characterization of Cyp2d22, a novel cytochrome P450 expressed in mouse mammary cells. Arch Biochem Biophys. 2000;381:191-204.
5. Chaluvadi MR, Kinloch RD, Nyagode BA, Richardson TA, Raynor MJ, Sherman M, et al. Regulation of hepatic cytochrome P450 expression in mice with intestinal or systemic infections of citrobacter rodentium. Drug Metab Dispos. 2009;37:366-74.
6. Merrell MD, Nyagode BA, Clarke JD, Cherrington NJ, Morgan ET. Selective and cytokine-dependent regulation of hepatic transporters and bile acid homeostasis during infectious colitis in mice. Drug Metab Dispos. 2014;42:596-602.
7. Zhang J, Chaluvadi MR, Reddy R, Motika MS, Richardson TA, Cashman JR, et al. Hepatic flavin-containing monooxygenase gene regulation in different mouse inflammation models. Drug Metab Dispos. 2009;37:462-8.
8. Enokizono J, Kusuhara H, Sugiyama Y. Regional expression and activity of breast cancer resistance protein (Bcrp/Abcg2) in mouse intestine: overlapping distribution with sulfotransferases. Drug Metab Dispos. 2007;35:922-8.
9. Takahashi S, Sakakibara Y, Mishiro E, Kouriki H, Nobe R, Kurogi K, et al. Molecular cloning, expression, and characterization of mouse amine N-sulfotransferases. Biochem Biophys Res Commun. 2008;375:531-5.
10. Richardson TA, Sherman M, Kalman D, Morgan ET. Expression of UDP-glucuronosyltransferase isoform mRNAs during inflammation and infection in mouse liver and kidney. Drug Metab Dispos. 2006;34:351-3.
11. Haque MW, Bose P, Siddique MUM, Sunita P, Lapenna A, Pattanayak SP. Taxifolin binds with LXR (alpha & beta) to attenuate DMBA-induced mammary carcinogenesis through mTOR/Maf-1/PTEN pathway. Biomed Pharmacother. 2018;105:27-36.
12. Penna F, Busquets S, Toledo M, Pin F, Massa D, Lopez-Soriano FJ, et al. Erythropoietin administration partially prevents adipose tissue loss in experimental cancer cachexia models. J Lipid Res. 2013;54:3045-51.
13. Overbergh L, Giulietti A, Valckx D, Decallonne R, Bouillon R, Mathieu C. The use of real-time reverse transcriptase PCR for the quantification of cytokine gene expression. J Biomol Tech. 2003;14:33-43.
14. Grabie N, Hsieh DT, Buono C, Westrich JR, Allen JA, Pang H, et al. Neutrophils sustain pathogenic CD8+ T cell responses in the heart. Am J Pathol. 2003;163:2413-20.
15. Baudino L, Yoshinobu K, Morito N, Kikuchi S, Fossati-Jimack L, Morley BJ, et al. Dissection of genetic mechanisms governing the expression of serum retroviral gp70 implicated in murine lupus nephritis. J Immunol. 2008;181:2846-54.

16. Lan JG, Cruickshank SM, Singh JC, Farrar M, Lodge JP, Felsburg PJ, et al. Different cytokine response of primary colonic epithelial cells to commensal bacteria. World J Gastroenterol. 2005;11:3375-84.
17. Shirai R, Hirano F, Ohkura N, Ikeda K, Inoue S. Up-regulation of the expression of leucine-rich alpha(2)-glycoprotein in hepatocytes by the mediators of acute-phase response. Biochem Biophys Res Commun. 2009;382:776-9.
18. Mimche PN, Brady LM, Bray CF, Lee CM, Thapa M, King TP, et al. The receptor tyrosine kinase EphB2 promotes hepatic fibrosis in mice. Hepatology. 2015;62:900-14.
