# Supplementary material for: A non-lethal malarial infection results in reduced drug metabolizing enzyme expression and drug clearance in mice
Source: Malar J. 2019 Jul 12;18:234. doi: 10.1186/s12936-019-2860-5 (PMC6624958; doi:10.1186/s12936-019-2860-5)
Supplement: Supplementary file 2 — Additional file 2: Table S2. Antibodies used for Western blotting. [file 12936_2019_2860_MOESM2_ESM.pdf]

Table S2. Antibodies used for Western blotting

| Mouse target proteins | Antigen used to raise antibodies | Type | Species | Dilution | Source | Catalog number | Reference |
|---|---|---|---|---|---|---|---|
| Cyp2b | CYP2B1 | polyclonal | rabbit | 1:5000 | James Halpert, University of Connecticut | N/A | (1) |
| Cyp2c | CYP2C11 | polyclonal | rabbit | 1:10,000 | In-house | N/A | (2) |
| Cyp2d | Cyp2d9 | polyclonal | rabbit | 1:5000 | Masahiko Negishi, NIEHS | N/A | (3) |
| Cyp2e1 | Rat CYP2E1 | polyclonal | Rabbit | 1:5000 | Magnus Ingelman-Sundberg, Karolinska Institute | N/A | (4) |
| Cyp3a | CYP3A1 | polyclonal | rabbit | 1:5000 | James Halpert, University of Connecticut | N/A | (5) |
| Cyp4a | CYP4A11 peptide | monoclonal | rabbit | 1:2000 | Abcam, Cambridge, MA | Ab-140635 | (6) |
| Gapdh | Rabbit GAPDH | monoclonal | mouse | 1:10,000 | Millipore, Temecula, CA | MAB374 | (7) |
| Nos2 | Mouse Nos2 | Polyclonal | rabbit | 1:2,000 | Santa Cruz Biotechnology, Inc, Santa Cruz, CA | Sc-650 | (6) |

## References

1. Duignan DB, Sipes IG, Leonard TB, Halpert JR. Purification and characterization of the dog hepatic cytochrome P-450 isozyme responsible for the metabolism of 2,2',4,4',5,5'-hexachlorobiphenyl. Arch Biochem Biophys. 1987;255:290-303.
2. Morgan ET, MacGeoch C, Gustafsson JA. Sexual differentiation of cytochrome P-450 in rat liver. Evidence for a constitutive isozyme as the male-specific 16 alpha-hydroxylase. Mol Pharmacol. 1985;27:471-9.
3. Devore K, Harada N, Negishi M. Characterization of testosterone 16 alpha-hydroxylase (I-P-450(16) alpha) induced by phenobarbital in mice. Biochemistry. 1985;24:5632-7.
4. Johansson I, Ekstrom G, Scholte B, Puzycki D, Jornvall H, Ingelman-Sundberg M. Ethanol-, fasting-, and acetone-inducible cytochromes P-450 in rat liver: regulation and characteristics of enzymes belonging to the IIB and IIE gene subfamilies. Biochemistry. 1988;27:1925-34.

5. Graves PE, Kaminsky LS, Halpert J. Evidence for functional and structural multiplicity of pregnenolone-16 alpha-carbonitrile-inducible cytochrome P-450 isozymes in rat liver microsomes. Biochemistry. 1987;26:3887-94.
6. Mimche SM, Nyagode BA, Merrell MD, Lee CM, Prasanphanich NS, Cummings RD, et al. Hepatic cytochrome P450s, phase II enzymes and nuclear receptors are downregulated in a Th2 environment during Schistosoma mansoni infection. Drug Metab Dispos. 2014;42:134-40.
7. Lee CM, Tripathi S, Morgan ET. Nitric oxide-regulated proteolysis of human CYP2B6 via the ubiquitin-proteasome system. Free Radic Biol Med. 2017;108:478-86.
