# Supplementary material for: A non-lethal malarial infection results in reduced drug metabolizing enzyme expression and drug clearance in mice
Source: Malar J. 2019 Jul 12;18:234. doi: 10.1186/s12936-019-2860-5 (PMC6624958; doi:10.1186/s12936-019-2860-5)
Supplement: Supplementary file 3 — Additional file 3: Fig. S1. Standard curves for caffeine, buproprion, tolbutamide, midazolam and bufuralol measured by HILIC/MS. [file 12936_2019_2860_MOESM3_ESM.pdf]

Mimche, SM et al. A nonlethal malarial infection results in reduced drug metabolizing enzyme expression and drug clearance in mice. Fig. S1.

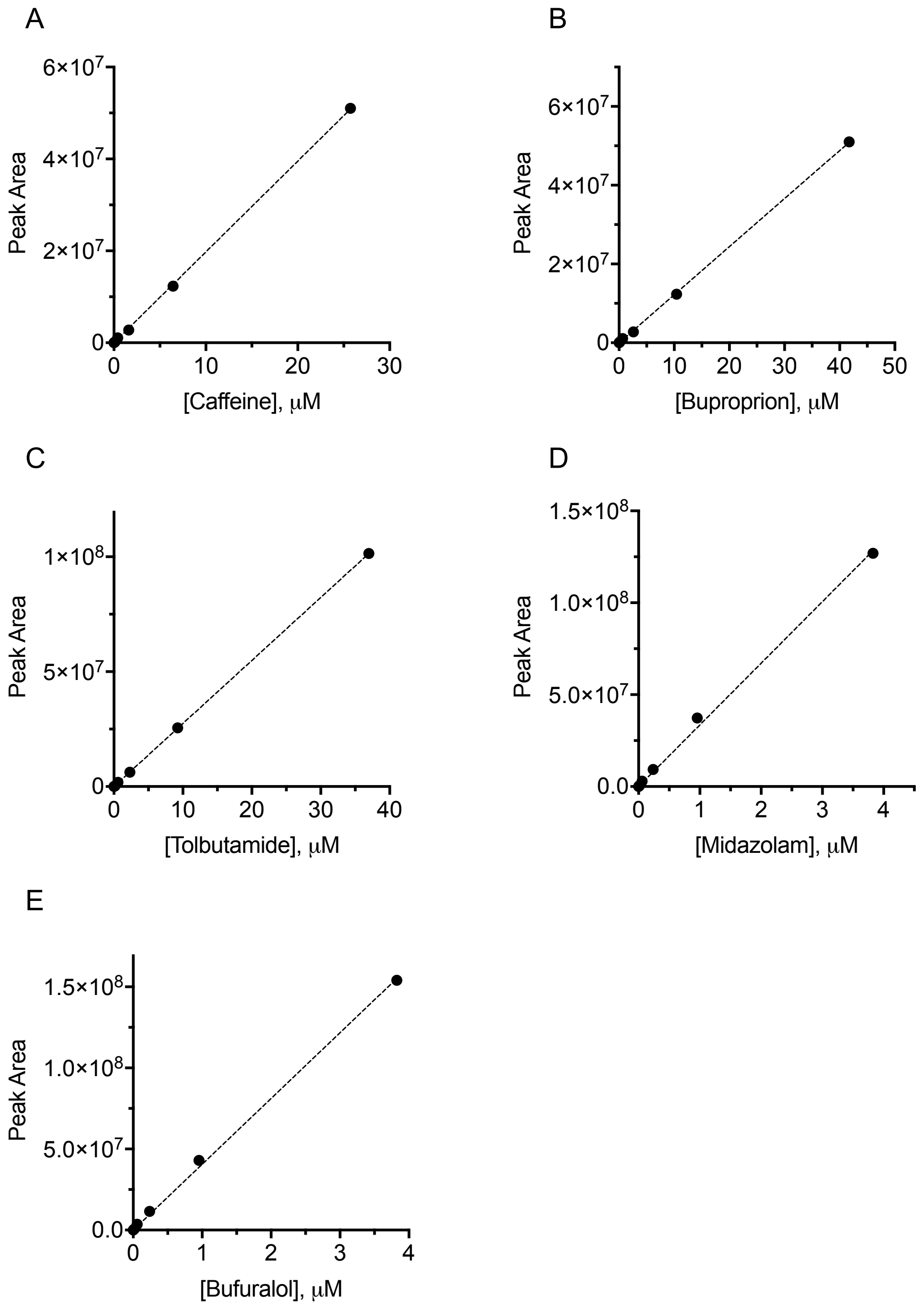

Figure S1. Standard curves for caffeine, buproprion, tolbutamide, midazolam and bufuralol measured by HILIC/MS. The drugs were spiked into naïve blood and processed identically to the experimental samples.
