# Supplementary material for: A non-lethal malarial infection results in reduced drug metabolizing enzyme expression and drug clearance in mice
Source: Malar J. 2019 Jul 12;18:234. doi: 10.1186/s12936-019-2860-5 (PMC6624958; doi:10.1186/s12936-019-2860-5)
Supplement: Supplementary file 6 — Additional file 6: Fig. S2. Additional analyses of mRNA expression in mouse livers. [file 12936_2019_2860_MOESM6_ESM.pdf]

Mimche, SM et al. A nonlethal malarial infection results in reduced drug metabolizing enzyme expression and drug clearance in mice. Fig. S2.

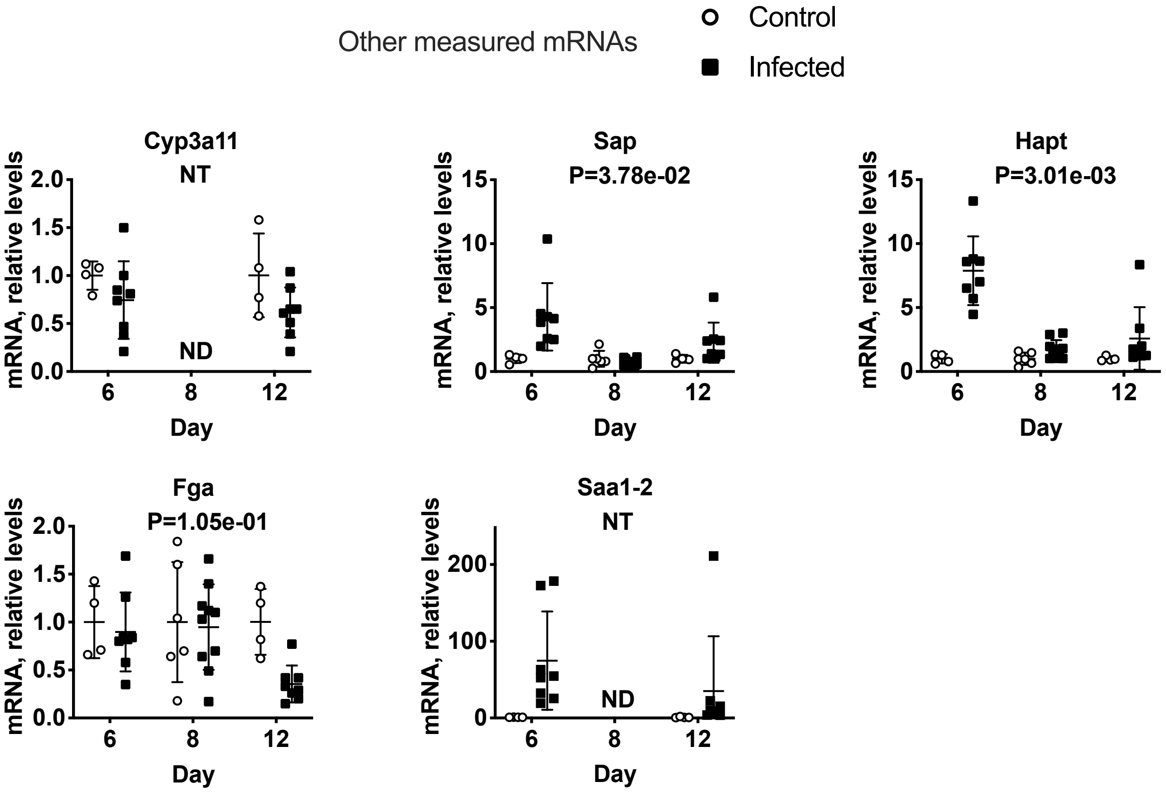

Figure S2. Additional analyses of mRNA expression in mouse livers. Mice were euthanized at 6, 8 and 12 days after infection with *Pcc*AS and their livers analyzed by RT-qPCR for mRNA expression of the designated genes. The data are the average of two separate experiments. Values are mean ± SD, and expression in naive mice was set at 1. Differences in mRNA expression between control and infected mice among the groups were analyzed by MANOVA with Bonferroni corrected one-way ANOVAs as a post-hoc test. *P<1.47e-03, **P<7.35e-04, ***P<1.47e-05, significantly different from naïve group. NT, not tested for significance; ND, not determined. For days 6, 8 and 12, n= 4, 6 and 4 in the naïve groups and n= 8, 10 and 8 in the infected groups, respectively.
