# Supplementary material for: A non-lethal malarial infection results in reduced drug metabolizing enzyme expression and drug clearance in mice
Source: Malar J. 2019 Jul 12;18:234. doi: 10.1186/s12936-019-2860-5 (PMC6624958; doi:10.1186/s12936-019-2860-5)
Supplement: Supplementary file 7 — Additional file 7: Fig. S3. Effects of P. chabaudi AS infection on mRNA expression of LXR target genes in mouse livers. [file 12936_2019_2860_MOESM7_ESM.pdf]

Mimche, SM et al. A nonlethal malarial infection results in reduced drug metabolizing enzyme expression and drug clearance in mice. Fig. S3.

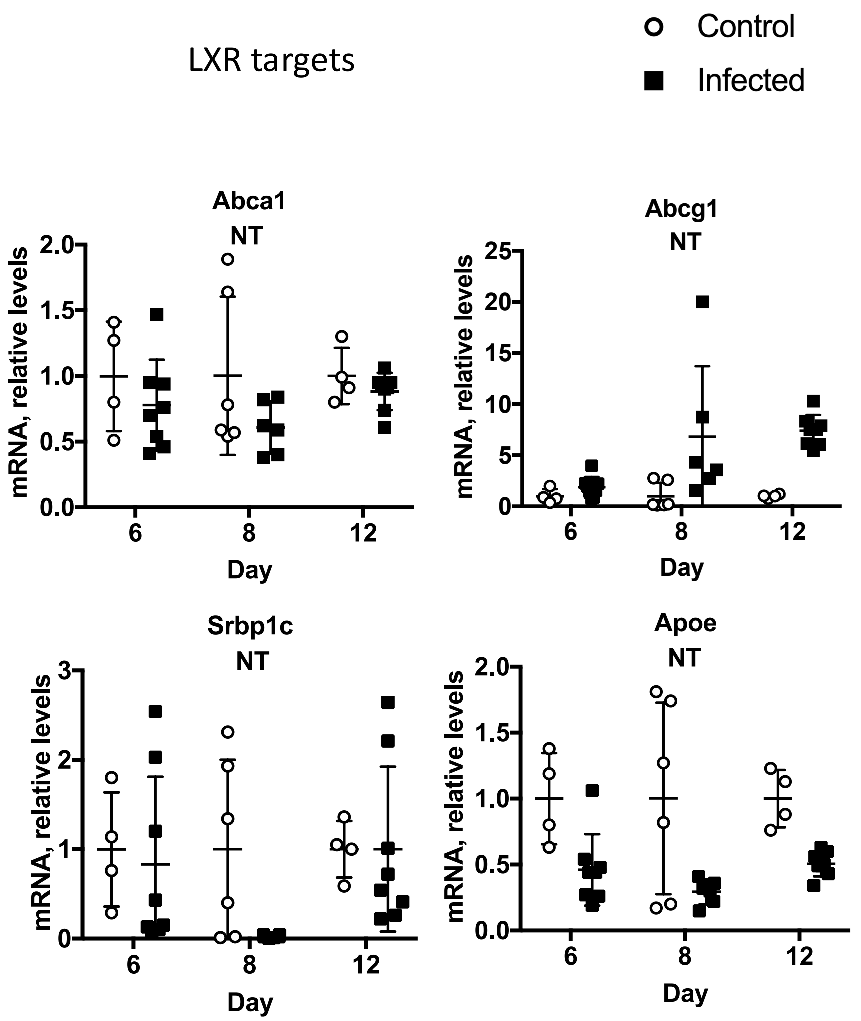

Figure S3. Effects of *Pcc*AS infection on mRNA expression of LXR target genes in mouse livers. Mice were euthanized at 6, 8 and 12 days after infection with *Pcc*AS and their livers analyzed by RT-qPCR for mRNA expression of LXR genes. The data are the average of two separate experiments. Values are mean ± SD, and expression in naive mice was set at 1. NT, not tested for significance. For days 6, 8 and 12, n= 4, 6 and 4 in the naïve groups and n= 8, 10 and 8 in the infected groups, respectively.
